# Germ Cell-Specific Targeting of DICER or DGCR8 Reveals a Novel Role for Endo-siRNAs in the Progression of Mammalian Spermatogenesis and Male Fertility

**DOI:** 10.1371/journal.pone.0107023

**Published:** 2014-09-22

**Authors:** Céline Zimmermann, Yannick Romero, Maria Warnefors, Adem Bilican, Christelle Borel, Lee B. Smith, Noora Kotaja, Henrik Kaessmann, Serge Nef

**Affiliations:** 1 Department of Genetic Medicine and Development, University of Geneva Medical School, Geneva, Switzerland; 2 Center for Integrative Genomics, University of Lausanne and Swiss Institute of Bioinformatics, Lausanne, Switzerland; 3 MRC Centre for Reproductive Health, University of Edinburgh, The Queen's Medical Research Institute, Edinburgh, United Kingdom; 4 Department of Physiology, Institute of Biomedicine, University of Turku, Turku, Finland; Cardiff University, United Kingdom

## Abstract

Small non-coding RNAs act as critical regulators of gene expression and are essential for male germ cell development and spermatogenesis. Previously, we showed that germ cell-specific inactivation of *Dicer1*, an endonuclease essential for the biogenesis of micro-RNAs (miRNAs) and endogenous small interfering RNAs (endo-siRNAs), led to complete male infertility due to alterations in meiotic progression, increased spermatocyte apoptosis and defects in the maturation of spermatozoa. To dissect the distinct physiological roles of miRNAs and endo-siRNAs in spermatogenesis, we compared the testicular phenotype of mice with *Dicer1* or *Dgcr8* depletion in male germ cells. *Dgcr8* mutant mice, which have a defective miRNA pathway while retaining an intact endo-siRNA pathway, were also infertile and displayed similar defects, although less severe, to *Dicer1* mutant mice. These included cumulative defects in meiotic and haploid phases of spermatogenesis, resulting in oligo-, terato-, and azoospermia. In addition, we found by RNA sequencing of purified spermatocytes that inactivation of *Dicer1* and the resulting absence of miRNAs affected the fine tuning of protein-coding gene expression by increasing low level gene expression. Overall, these results emphasize the essential role of miRNAs in the progression of spermatogenesis, but also indicate a role for endo-siRNAs in this process.

## Introduction

Spermatogenesis is the development of mature haploid spermatozoa, and ensures continuous gamete production throughout adult life. It is divided into three distinctive phases: First, spermatogonia proliferate to give rise to primary spermatocytes, which then undergo two meiotic divisions leading to the production of haploid spermatids. The final phase of the process, spermiogenesis, involves the maturation and final morphological transformation of spermatids into mature spermatozoa [1].

Spermatogenesis is a complex biological process governed by phase-specific gene expression programs that are tightly controlled at both the transcriptional and post-transcriptional level [2]. Growing evidence suggests that small non-coding RNAs (sncRNAs) are important regulators of gene expression, and mainly function at the post-transcriptional level by affecting mRNA stability, turnover, processing, storage and translation [3]. These sncRNAs can be classified into different categories based on their biogenesis, mechanism of action and function. Male germ cells have been reported to express not only microRNAs (miRNAs) [4] but also endogenous small interfering RNAs (endo-siRNAs) [5] and piwi-interacting RNAs (piRNAs) [6], [7], [8]. Hundreds of miRNAs are expressed in mammalian testes and male germ cells [9]. Canonical miRNAs are initially transcribed as pri-miRNAs, which are recognized in the nucleus by the dsRNA-binding protein DGCR8 and processed by the RNase III endonuclease DROSHA into 70-nucleotide pre-miRNAs [10], [11]. These are then exported into the cytoplasm and further cleaved by DICER1, another RNase III endonuclease, to produce 21–25 nucleotide small dsRNAs [12]. These mature miRNAs are subsequently incorporated into RNA-induced silencing complexes (RISC). They act as sequence guides to mediate sequence-specific binding of the RISCs to their target mRNAs, and direct either their translational repression and/or degradation [13], [14]. In addition, male germ cells also express numerous endo-siRNAs, which are derived from naturally occurring double stranded precursors, but their function in regulating spermatogenesis remains unclear [5]. While the biogenesis of these testicular endo-siRNAs requires DICER1, it does not involve DROSHA/DGCR8 [5].

Mouse models with the conditional deletion of *Dicer1* in the male germ line have demonstrated the importance of sncRNAs in primordial germ cell development [15], [16] and spermatogenesis [17], [18], [19], [20]. We have previously shown that the spermatogonia-specific deletion of *Dicer1*, using Cre under the control of the *Ddx4* promoter, leads to infertility [18]. This is due to severe cumulative defects during the meiotic and spermiogenic phases of spermatogenesis which eventually result in the absence of functional spermatozoa. These defects lead to delayed progression of meiotic prophase I and massive apoptosis of spermatocytes. The transition from round spermatids to mature spermatozoa was also severely affected since the few spermatozoa that did form were immobile and misshapen, exhibiting irreversible morphological defects due to disturbances in acrosome formation and nuclear condensation. Interestingly, less severe reproductive phenotypes were observed when *Dicer1* was inactivated in male germ cells at later developmental stages [19], [20]. The more severe phenotype observed with the *Ddx4:Cre* transgene likely represents the sum of low impact defects that accumulate at various meiotic and post-meiotic stages.

However, these studies leave essential questions unanswered regarding the role of small RNA pathways on the germ cell transcriptome and the regulation of protein-coding mRNAs during spermatogenesis. In addition, since DICER1 is required for the processing of both miRNAs and endo-siRNAs, it is important to clarify the functional involvement of these two sncRNA species at each step of spermatogenesis. In this study, our goal was firstly to define the role played by the miRNA and endo-siRNA pathways in mediating spermatogenic processes and male fertility, by comparing the phenotypic differences of mice lacking either *Dicer1* or *Dgcr8* in the male germ lineage. Secondly, we aimed to characterize the global changes in protein-coding gene expression by RNA sequencing in spermatocytes lacking *Dicer1*.

## Results

### Selective inactivation of *Dicer1* and *Dgcr8* in male germ cells

**Figure 1:**
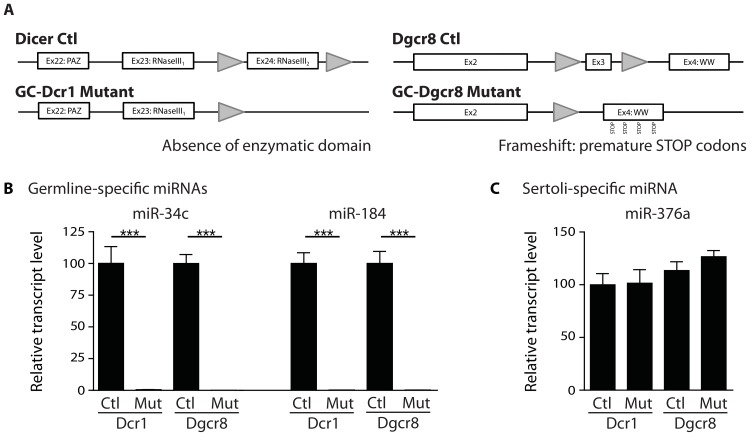
Germ cell-specific depletion of miRNAs in GC-Dcr1 and GC-Dgcr8 mutant testes. (A) Schematic representation of *Dicer1* and *Dgcr8* loci. Exon 24 of the *Dicer1* gene is flanked with loxP sites (grey triangles) and excision occurs upon *Ddx4-Cre* recombinase expression leading to loss of the RNaseIII domain 2. Excision of exon 3 of the *Dgcr8* locus leads to a sequence frameshift and introduces multiple premature STOP codons. (B) Histograms showing the absence of expression of spermatocyte-specific miRNAs in GC-Dgcr8 and GC-Dcr1 mutant total testes. In contrast, expression of Sertoli cell-specific miRNAs in both mutants was not affected (C). Results are mean ± SEM, * = p<0.05, ** = p<0.005, *** = p<0.0001 GC-Dcr1 mutant vs. control, GC-Dgcr8 mutant vs. control or GC-Dcr1 vs. GC-Dgcr8.

To dissect and characterize the physiological role of miRNAs and endo-siRNAs in spermatogenesis, we compared the phenotype of mice deficient in either *Dicer1* or *Dgcr8* in male germ cells. Specific inactivation of *Dicer1* and *Dgcr8* in the male germ cell lineage was achieved by crossing mice bearing two loxP-flanked alleles of *Dicer1* (*Dcr1^fx/fx^*) [21] or *Dgcr8* (*Dgcr8^fx/fx^*) [22] with a *Ddx4* (MVH/Vasa) promoter-driven transgenic Cre line [23]. The *Ddx4-Cre* transgene has been shown to induce Cre-mediated recombination in >90% and >97% of spermatogonia by embryonic day (E) 18 and postnatal day (P) 3, respectively [23]. For simplicity, germ cell-specific deletion of *Dicer1* (*Dcr1^fx/fx^;Ddx4:Cre*) and *Dgcr8* (*Dgcr8^fx/fx^;Ddx4:Cre*) is abbreviated as GC-Dcr1 and GC-Dgcr8, respectively. Loss of function was achieved by Cre-mediated excision of exon 24 in the *Dicer1* locus, which results in a loss of RNAse III enzymatic activity in GC-Dcr1 mutant males. In GC-Dgcr8 mutant males, deletion of exon 3 of *Dgcr8* leads to a frameshift, resulting in multiple premature stop codons (**Fig. 1A**). Importantly we showed, by means of qRT-PCR in both GC-Dcr1 and GC-Dgcr8 P60 mutant testes, a complete loss of miR-34c and miR-184, two miRNAs expressed specifically in spermatocytes/spermatids [24], [25] (**Fig. 1B**). On the other hand, miRNAs specifically expressed in Sertoli cells [26] were not affected (**Fig. 1C**) [19]. We conclude that Cre-mediated deletion of *Dicer1* and *Dgcr8* was fully penetrant and resulted in a complete and specific loss of miRNA biogenesis in the male germ-cell lineage

### Loss of endo-siRNAs from germ cells increases the severity of the testicular phenotype

**Figure 2:**
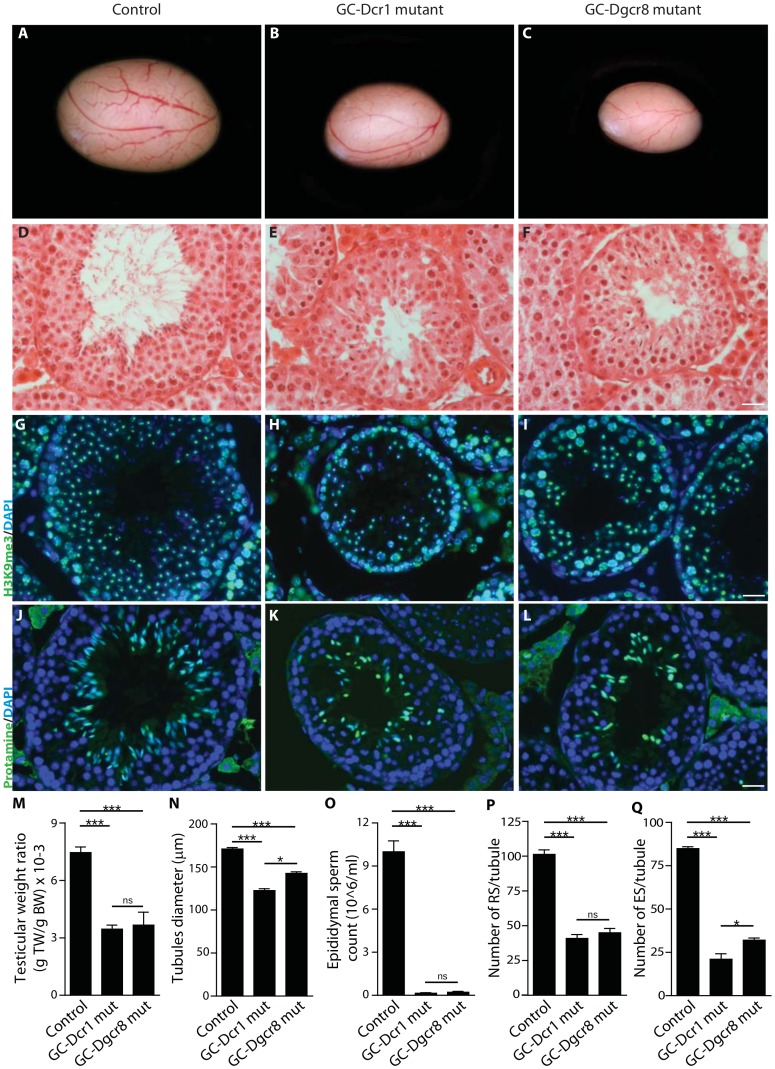
Reduction in testis size and near complete absence of mature spermatozoa in GC-Dcr1 and GC-Dgcr8 mutant mice. (A–C, M) At P60, testis weight showed a 55% and 50% reduction in GC-Dcr1 (n = 16) and GC-Dgcr8 mutants (n = 5) compared to control testes (n = 17). H&E staining of testis sections (D–F) revealed several defects in the architecture of the seminiferous epithelium, near complete absence of mature spermatozoa, and reduced tubular diameter (N) (Control: 170.9 µm^2^±1.7, GC-Dcr1 mut: 122.5 µm^2^±2.2, GC-Dgcr8 mut: 142.6 µm^2^±1.8). (G–I) Anti-H3K9me3 stained sex-body in round spermatids (RS). We observed a 60% reduction in the number of RS per tubule in GC-Dcr1 mutants and 55% reduction in GC-Dgcr8 mutants compared to control testes (P). (J–L) Anti-protamine revealed a 75% reduction in the number of elongated spermatids (ES) per tubule in GC-Dcr1 mutants and a 62% reduction in GC-Dgcr8 mutants compared to control testes (Q). DAPI (blue) was used for nuclear staining. (O) Epididymal sperm count analysis showed a 99% decrease in GC-Dcr1 mutants and 96% in GC-Dgcr8 mutants. TW: Testis Weight, BW: Body Weight, RS: Round Spermatids, ES: Elongated Spermatids. Results are mean ± SEM, * = p<0.05, ** = p<0.005, *** = p<0.0001 GC-Dcr1 mutant vs. control, GC-Dgcr8 mutant vs. control or GC-Dcr1 vs. GC-Dgcr8. Scale bars: 20 µm (D–L).

At adulthood (P60), GC-Dcr1 mutant mice displayed reduced testis size (**Fig. 2A–C**) and a 55% decrease in testis weight compared to the control littermates. This was more severe than the 50% decrease observed in GC-Dgcr81 mutants, however not statistically significant (**Fig. 2M**). There was no difference in seminal vesicles and plasma testosterone levels between the three groups (**Table S1**). Fertility tests revealed that both GC-Dcr1 and GC-Dgcr8 mutant mice were infertile (data not shown). Histological analysis of P60 testis indicated that the seminiferous epithelium was drastically altered in both GC-Dcr1 and GC-Dgcr8 mutants compared to control mice (**Fig. 2D–F**). However, when analyzing in details the testicular phenotype, we found that the severity of the phenotype was consistently more severe in GC-Dcr1 compared to GC-Dgcr8 mutant animals.

The diameter of seminiferous tubules was decreased in both mutants, although significantly more so in GC-Dcr1 (−29%) than in GC-Dgcr8 (−17%) mutant mice (**Fig. 2N**). Overall, the alteration in spermatogenesis results in a massive reduction in epididymal sperm count in both GC-Dcr1 and GC-Dgcr8 mutants, by ∼99% and ∼96% respectively (**Fig. 2O**). Indeed, mature spermatozoa were rarely found in the tubule lumens and the epididymal ducts (**Fig. 2D–F** and data not shown), due to a strong decrease of round and elongated spermatid numbers in both mutants as observed in immunofluorescence (IF) staining of H3K9me3 and protamine respectively (**Fig. 2G–L and P–Q**). The H3K9me3 protein is present on all chromosomes in spermatocytes, while it is restricted to the sexual chromosomes in round spermatids. The protamine is a specific protein of elongated spermatids. Again, the number of elongated spermatids was significantly more decreased in GC-Dcr1 (−75%) than in GC-Dgcr8 (−62%) mutant testes. The results indicate that germ cell-specific deletion of *Dicer1* or *Dgcr8* resulted in reduced testis size and sperm count, and male infertility. However, the severity of the phenotype was slightly but consistently more severe in GC-Dcr1 mutant testes as exemplified by testis weight, the diameter of seminiferous tubules and number of spermatids or spermatozoa.

### Alterations in spermatogenesis appear as early as P15 in both GC-Dcr1 and GC-Dgcr8 mutants but germ cell apoptosis is more severe in GC-Dcr1 mutants

**Figure 3:**
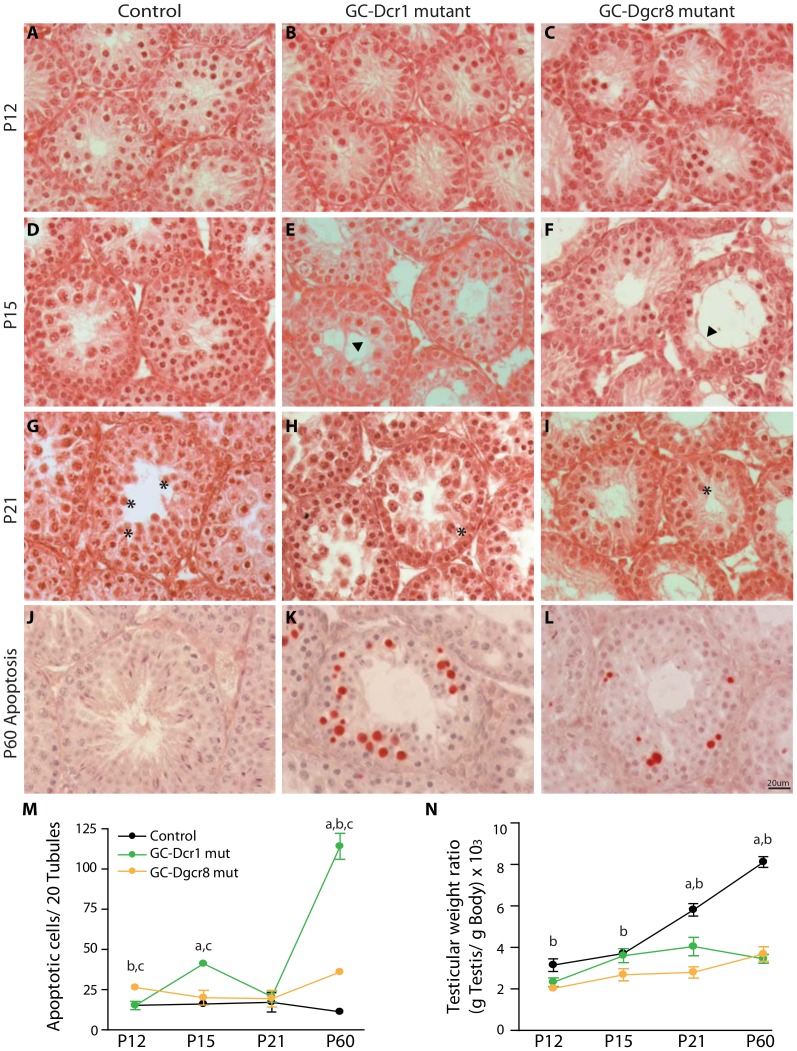
Tubular defects in GC-Dcr1 and GC-Dgcr8 mutant testes appear from P15. H&E staining of control (A,D,G), GC-Dcr1 mutant (B,E,H) and GC-Dgcr8 mutant (C,F,J) testes at P12 (A–C), P15 (D–F) and P21 (G–J). The anatomical defects include germ cells sloughing, Sertoli cell cytoplasmic extensions (arrowheads), reduction in the number of round spermatids (asterisks), and apoptosis and disorganization of the seminiferous epithelium. The increase of apoptotic rate (M) correlates with the reduced testis weight ratio (N) during the first wave of spermatogenesis. (J–L) Apoptotic cells revealed by TUNEL assay on testis sections at P60. Results are mean ± SEM (n = 3/genotype), a = p<0.05 GC-Dcr1 mut vs. control, b = p<0.05 GC-Dgcr8 mut vs. control and c = p<0.05 GC-Dcr1 mut vs. GC-Dgcr8 mut. Scale bars: 20 µm.

To further characterize the alterations in spermatogenesis that lead to infertility, we compared the testicular development of control, GC-Dcr1 and GC-Dgcr8 mutant animals from P12 to P21 – the early phases of the first spermatogenic wave. At P12, the testes of GC-Dcr1, GC-Dgcr8 and control animals were morphologically indistinguishable (**Fig. 3A–C**). In both GC-Dcr1 and GC-Dgcr8 mutants, the first abnormalities began to appear at P15, with germ cell sloughing and the appearance of Sertoli cell cytoplasmic extensions (arrowheads) (**Fig. 3D–F**). At P21, control mice had completed meiosis II and generated round spermatids (asterisks); however, these cells were scarce in GC-Dgcr8 mutants and almost completely absent in GC-Dcr1 animals (**Fig. 3G–I**).

We have previously shown that the loss of *Dicer1* significantly reduces the number of post-meiotic germ cells due to a massive apoptotic wave starting around P15 (**Fig. 3M**). We therefore investigated and compared germ cell survival in the context of *Dicer1* or *Dgcr8* depletion. A TUNEL assay revealed that while apoptosis was 3-times more elevated of control levels in GC-Dgcr8 mutants, this compares to 10-times more elevation in GC-Dcr1 mutant mice at P60 (GC-Dgcr8 mutant 35.8±1.7 apoptotic cells/20 tubules; GC-Dcr1 mutant 114.5±8.1 apoptotic cells/20 tubules; control 11.0±1.8 apoptotic cells/20 tubules **Fig. 3J–L, M**). As previously observed in GC-Dcr1 mutants, apoptotic cells in GC-Dgcr8 mutants were concentrated in some seminiferous tubules rather than being evenly distributed (**Fig. 3J–L**). Interestingly, the apoptotic wave in GC-Dgcr8 mutants occurred earlier than in GC-Dcr1 mutants, starting at P12 and then decreasing (**Fig. 3M**). Testis weight was also evaluated during this period and it was found to be reduced in GC-Dgcr8 mutants as early as P12 (**Fig. 3N**), an observation that nicely reflected the earlier onset of apoptosis in these animals.

### GC-Dcr1 mutants show stronger meiotic defects compared to GC-Dgcr8 mutants

**Figure 4:**
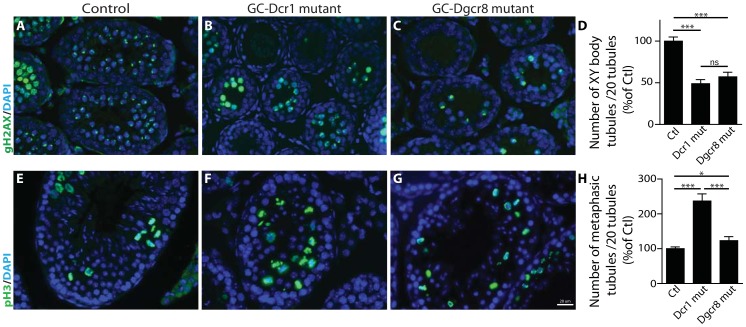
Meiotic defects in GC-Dcr1 and GC-Dgcr8 mutant testes. (A–D) Anti-γH2AX (green), present in the entire nucleus in early meiotic stages and located to the XY body during the pachytene stage, indicates a decreased number of tubules containing XY body positive cells in GC-Dcr1 and GC-Dgcr8 mutant testes compared to controls at P12. (E–H) Anti-pH3 (green), present in metaphasic cells, reveals a reduction in the number of metaphasic tubules within GC-Dcr1 and GC-Dgcr8 mutants in P60 testes compared to controls. DAPI (blue) was used for nuclear staining. Results are mean ± SEM (n = 3/genotype), * = p<0.05, ** = p<0.005, *** = p<0.0001 GC-Dcr1 mutant vs. control, GC-Dgcr8 mutant vs. control or GC-Dcr1 vs. GC-Dgcr8. Scale bars: 20 µm.

Since the first spermatogenic defects in both mutants are observed during the first apoptotic wave at around P15, the pachytene stage, we hypothesized that the zygotene to pachytene transition might be defective. We therefore investigated the levels of serine 139-phosphorylated γH2AX (γH2AX) at P12, during the zygotene-to-pachytene transition, in both GC-Dcr1 and GC-Dgcr8 mutant animals. In normal conditions, phosphorylation of γH2AX is associated with the repair of DNA double-strand breaks. Phosphorylated γH2AX is localized on all chromosomes during the leptotene and zygotene phases, but is found solely on the XY body (sex body) during the pachytene stage, when X- and Y-linked genes undergo transcriptional silencing. We found a ∼2-fold reduction in the number of pachytene-stage γH2AX-positive spermatocyte-containing tubules in both GC-Dgcr8 and GC-Dcr1 mutant mice, when compared to control mice (**Fig. 4A–D**). In contrast, when we used ser-10 phosphorylation of histone 3 as a specific marker of cells in metaphase (*i.e.* spermatogonia and late meiotic cells) we observed a significantly higher number of germ cells in meiotic metaphase in the seminiferous tubules of GC-Dcr1 animals compared to GC-Dgcr8 mutant and control mice at P60 (**Fig. 4E–H**).

Taken together, these findings indicate that *Dicer1* and *Dgcr8* depletions in germ cells have broadly similar deleterious effects on the first spermatogenic wave: they both partially block spermatocytes in late prophase I concomitant with an increase in apoptosis, although the defects were less severe in the GC-Dgcr8 mutant. These results confirm the essential role played by miRNAs during the first meiotic prophase, although the differences in the severity of the phenotypes also suggest a role for endo-siRNAs in this process.

### Impairment in spermiogenesis is more severe in GC-Dcr1 as compared to GC-Dgcr8 mutant mice

**Figure 5:**
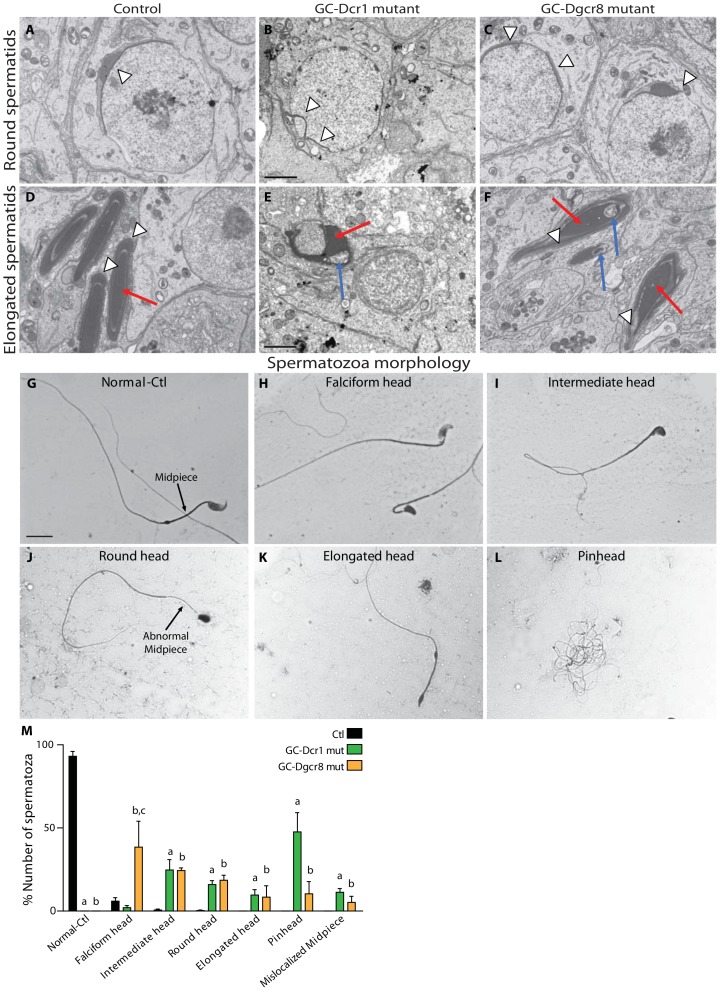
Impaired spermiogenesis leads to altered morphology of spermatozoa in both GC-Dcr1 and GC-Dgcr8 mutants at P60. Representative transmission electron micrographs from P60 control (A, D), GC-Dcr1 mutants (B,E) and GC-Dgcr8 mutants (C,F). In round spermatids (A–C), the acrosome is fragmented or asymmetric in both mutants (arrowheads). In elongated spermatids (D–F), nuclear shape (red arrows), chromatin condensation and the acrosome (arrowheads) are abnormal in both GC-Dcr1 and GC-Dgcr8 mutants. Note the presence of vacuoles within nuclei (blue arrows) in both mutants. Scale bar: 2 µm. H&E staining of epididymal sperm spreads of control (G) and mutant (H–L) adult mice. In contrast to control mice, spermatozoa of both mutant mice exhibited multiple defects of morphology, such as falciform head (H), intermediate head (I), round head (J), elongated head (K), pinhead (L) and abnormal midpiece (J). Scale bars: 10 µm. The histogram shows the percentage of spermatozoa in each category (M). Results are mean ± SEM (minimum n = 3/genotype), a = p<0.005 GC-Dcr1 mut vs. control, b = p<0.005 GC-Dgcr8 mut vs. control and c = p<0.005 GC-Dcr1 mut vs. GC-Dgcr8 mut.

Small non-coding RNAs are essential for the haploid phase of spermatogenesis. To assess the contribution of canonical miRNAs and endo-siRNAs in mediating spermiogenesis, we compared phenotypic differences between GC-Dcr1 and GC-Dgcr8 mutant mice. Using electron microscopy with P60 testes sections, we observed similar defects with variable severity in the spermatids of GC-Dcr1 and GC-Dgcr8 mice. First, the acrosome of round spermatids was fragmented and asymmetric, in both GC-Dgcr8 and GC-Dcr1 mutants (**Fig. 5A–C**, white arrow heads). Second, elongated spermatids exhibited defects at the chromatin condensation level with nuclear vacuoles (blue arrows, **Fig. 5E,F**) and displayed an abnormal head shape (compare red arrows between **Fig. 5D** **and** **Fig. 5E&F**). Although present in both mutants, these defects in chromatin compaction and abnormal head shape were more pronounced in GC-Dcr1 mice.

Since the round and elongated spermatids of mutants displayed severe morphological alterations, we further analyzed the morphology of the few remaining epididymal sperm cells (**Fig. 5G–M**). The vast majority of GC-Dcr1 mutant spermatozoa displayed phenotypic alterations similar to those observed in the GC-Dgcr8 mutants, but again the severity of the morphological defects was more severe. Normal spermatozoa were only found in control mice (**Fig. 5G, M**). In contrast, spermatozoa found in the epididymides of GC-Dcr1 and GC-Dgcr8 mutant individuals at P60 displayed variable morphological defects of the head and flagellum, which could be classified in different categories based on the severity of the phenotype. They range from falciform head shape spermatozoa (**Fig. 5H**), through intermediate head shape spermatozoa (**Fig. 5I**), round head shape spermatozoa (**Fig. 5J**), and elongated head shape spermatozoa (**Fig. 5K**) up to the most abnormal pinhead spermatozoa (**Fig. 5L**). Furthermore, we found abnormal midpiece in some spermatozoa in GC-Dcr1 and GC-Dgcr8 mutants (black arrow) (**Fig. 5J, M**). Interestingly, the type of spermatozoon-like structures found in the epididymides of GC-Dcr1 mice tend to display more severe phenotypes, with most spermatozoa showing the heaviest defect: the pinhead structure (**Fig. 5M**). In contrast, the spermatozoa found in GC-Dgcr8 mice usually exhibited milder defects and were very often categorized as falciform head shape.

These data confirm the essential role played by canonical miRNAs in the meiotic and haploid phases of spermatogenesis. However, the milder phenotypes observed in GC-Dgcr8 mutant testes suggest that endo-siRNAs also contribute to the regulation of spermatogenesis, although to a much lesser extent.

### Ablation of Dicer in germ cells causes large-scale alterations of the testicular transcriptome

**Figure 6:**
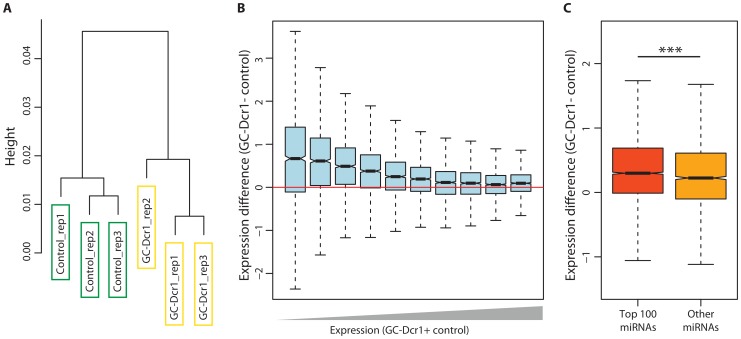
Altered mRNA transcriptome in GC-Dcr1 spermatocytes. (A) Hierarchical clustering of the six sequenced samples. We calculated the distance between each pair of samples as 1 - *rho*, where *rho* was the Spearman correlation coefficient for the gene expression levels in the two samples. Clustering was performed using the *hclust* function in R and Ward's method. (B) Overview of the normalized gene expression data. Genes were grouped into 10 equally sized bins based on their combined expression in GC-Dcr1 and control spermatocytes. Highly expressed genes are shown on the right. Expression change was calculated as the difference between the log_2_-transformed normalized expression values in GC-Dcr1 and control spermatocytes. (C) Comparison of expression change in genes targeted by highly and lowly expressed miRNAs (see text for details). Expression change was calculated as in (B).

To determine the role of small RNAs on the germ cell transcriptome and to further characterize the effects of Dicer1 deletion on the expression of protein-coding genes in male germ cells, we performed RNA-sequencing on isolated leptotene/pachytene spermatocytes from GC-Dcr1 mutant and control mice. Hierarchical clustering of the resulting gene expression data revealed that replicates from either GC-Dcr1 or control spermatocytes were more similar to each other than to other samples (**Fig. 6A**), implying that the deletion of Dicer1 caused considerable and reproducible changes in the transcriptome. Specifically, our analysis revealed a general upregulation of gene expression in GC-Dcr1 mutant spermatocytes (**Fig. 6B**), with a median expression level (normalized log2-transformed FPKM value; see Materials and Methods) of 2.37 in GC-Dcr1 spermatocytes and 2.15 in control spermatocytes (p<10^−15^, Wilcoxon signed rank test). The increased expression of protein-coding genes following *Dicer1* deletion is consistent with the expected lack of repressive regulation in miRNA-deficient cells [14], [27], [28].

### Targets of highly expressed miRNAs are preferentially upregulated in GC-Dcr1 spermatocytes

To further investigate the link between miRNA regulation and expression changes following *Dicer1* deletion, we considered the extent to which genes were targeted by the 100 most expressed miRNAs [29]. All genes that had been used for normalization were excluded from this analysis to avoid circularity. We found that genes targeted by many miRNAs were more likely to be highly upregulated in GC-Dcr1 spermatocytes (Spearman's correlation coefficient  = 0.092, p<10^−15^). The correlation, although significant, was relatively weak, which possibly could be due to the difficulties of predicting functional miRNA target sites. The association between the number of targeting miRNAs and the upregulation of gene expression in GC-Dcr1 spermatocytes suggests that many of the transcriptomic differences between mutant and control cells could be a direct effect of the loss of miRNA regulation. However, it is possible that other differences between targeted and non-targeted genes could explain this result. To control for this, we compared genes that were targeted by the 100 most expressed miRNAs, with genes targeted exclusively by lowly expressed miRNAs. This analysis showed that targets of highly expressed miRNAs were significantly more upregulated than other miRNA targets (**Fig. 6C**; median expression change: 0.30 and 0.22, p = 1.5×10^−8^, Mann-Whitney test), consistent with our hypothesis that many genes with increased expression in GC-Dcr1 spermatocytes are directly regulated by miRNAs.

Interestingly, we found that protein-coding gene affected in GC-Dcr1 spermatocytes were significantly enriched with numerous functional Gene Ontology (GO) categories among them regulation of programmed cell death which is consistent with our previous data showing apoptosis (see **Table S2** for a complete list of significantly enriched GO categories). It included genes involved in the canonical caspase-dependent apoptotic pathway such as the pro-apoptotic genes *Bax*, *Apaf1*, *CytC*, *Casp7* and *9* (see **Table S3**) [30]. Genes related to spermatogenesis or meiosis were not found to be highly upregulated in the GC-Dcr1 mutants spermatocytes (data not shown). Thus, we propose that the meiotic and spermiogenic defects observed in GC-Dcr1 mutant are not associated with altered expression of known spermatogenic or meiotic genes, but instead are caused by the upregulation of numerous lowly expressed genes, promoting apoptosis and affecting spermatogenesis at multiple stages.

## Discussion

Small non-coding RNAs, including endo-siRNAs and miRNAs, are abundantly expressed in male germ cells and play an important role as post-transcriptional regulators of gene expression. The present study aims to further characterize the molecular mechanisms that lead to massive apoptosis, spermatogenic failure and infertility following *Dicer1* inactivation in male germ cells.

In order to evaluate the contribution of canonical miRNAs and endo-siRNAs in mediating the meiotic and haploid phases of spermatogenesis, we first compared the phenotypes of GC-Dcr1 and GC-Dgcr8 mutant mice. Germ cells deficient in *Dicer1* lack both canonical miRNAs and endo-siRNAs, whereas those deficient in *Dgcr8* lack only the former. Interestingly, the ablation of either *Dicer1* or *Dgcr8* in the germ cell lineage using the *Ddx4:Cre* transgene led to complete infertility. We observed similar cumulative defects in meiotic and post-meiotic germ cells, ultimately resulting in a low number of non-functional and abnormal spermatozoa. This indicates that miRNAs are absolutely essential for germ cell development and spermatogenesis. However, the GC-Dgcr8 mutant phenotype seemed to be slightly less severe than in GC-Dcr1 mutants, suggesting a potential, lesser role for endo-siRNAs in the meiotic and post-meiotic stages of spermatogenesis. We found that several testicular parameters were less severely affected in GC-Dgcr8 mice, including reduction in testis weight (50% versus 55% for GC-Dcr1), in the diameter of seminiferous tubules (∼17% versus ∼28% for GC-Dcr1), in epididymal sperm count (∼96% versus ∼99% for GC-Dcr1), in apoptotic germ cells at P60 (∼5 fold lower for GC-Dgcr8), in spermatocytes blocked in metaphase (∼1.2 fold increase versus ∼2.4 fold increase for GC-Dcr1) and in morphological alterations of elongated spermatids and sperm cells.

The functional differences between miRNA and endo-siRNA pathways in spermatogenesis have also been investigated by comparing the phenotypes of mice lacking either *Dicer1* or *Drosha* in male germ cells [19]. This functional approach is similar to our experimental design but uses a *Drosha* floxed allele instead of a *Dgcr8* floxed allele to inactivate the microprocessor complex DROSHA-DGCR8, and a *Stra8:iCre* transgene instead of the *Ddx4:Cre* transgene to target male germ cell specific deletion. Similarly to the *Dicer1* mutant, ablation of *Drosha* in the male germ line results in complete infertility characterized by impaired spermatogenesis, and depletion of spermatocytes and spermatids, leading to oligozoospermia and azoospermia. However, comparison of the *Dicer1* and *Drosha* phenotypes revealed that spermatogenic disruption appeared to be more severe in *Drosha* mutants. In particular, the proportion of seminiferous tubules devoid of elongating and elongated spermatids was higher in *Drosha* mutant mice (∼23% versus ∼7% for *Dicer1* mutant testes). Similarly, seminiferous tubules containing vacuoles and multinucleated giant cells were also more frequent (∼43% in *Drosha* versus ∼24% in *Dicer1* mutant mice). These findings somewhat contradict our results, which show that deletion of *Dgcr8* in male germ cells resulted a slightly milder phenotype when compared to *Dicer1* mutant mice. This apparent discrepancy might be explained by the inactivation of different genes (*Dgcr8* versus *Drosha*), the use of different Cre-transgenic lines (*Ddx4:Cre* versus *Stra8:iCre*) and/or differences in the genetic background of the mouse strains. These differences in experimental design may affect both the penetrance and the developmental stage when floxed alleles are excised. More precisely, the *Ddx4:cre* transgene has been reported to induce specific Cre-mediated recombination in >90% and >97% of spermatogonia by embryonic day (E) 18 and postnatal day (P) 3, respectively [18], [23]. In contrast, expression of the *Stra8:iCre* transgene does not start in differentiating spermatogonia until P3 [31], and Cre-mediated deletion occurs at full penetrance only by P21 in spermatocytes and haploid germ cells [19]. Alternatively, it is possible that small RNA-independent functions of DROSHA could explain the more severe phenotype. Indeed, several studies demonstrated that DROSHA is implicated in mRNA cleavage, thus regulating their expression in a post-transcriptional manner [32], [33], [34], [35]. One of DROSHA's target is the *Dgcr8* itself, suggesting that DROSHA deletion affects DGCR8's action. Finally, it has been recently shown that DROSHA can bind to specific promoter-proximal regions of several human genes to regulate their expression in a transcription-dependent manner [36]. This process is not associated with the small RNA synthesis or mRNA cleavage function of DROSHA.

Aside from these variations in the severity of the testicular phenotype, all the results clearly point toward an essential role for the miRNA pathway in mediating both the meiotic and spermiogenic phases of spermatogenesis. However, the functional role of the endo-siRNA pathway in spermatogenesis remains unclear, although the milder phenotype observed in GC-Dgcr8 does appear to suggest a potential role for endo-siRNAs. A total of 73 endo-siRNAs have been identified by RNA sequencing in the mouse testis [5]. Among the predicted target transcripts, the most abundant were mRNAs (∼92%), with primary target sites located mostly in their 3′-UTRs (∼93%) [5]. Testicular endo-siRNAs have been shown to induce degradation of their mRNA targets *in vitro* suggesting that endo-siRNAs, like miRNAs, may regulate gene expression at the posttranscriptional level and have a potential role in spermatogenesis. Furthermore, as they display multiple hits on several chromosomes, endo-siRNAs may potentially have roles in chromatin modification and epigenetic regulation, as has been observed in other organisms [37], [38], [39], [40]. Interestingly, there is a stark contrast between the male germ line, where miRNAs play an essential role, and the female germ line. Although both miRNAs and endo-siRNAs are present in the developing oocytes [41], [42], *Dgcr8* mutant oocytes mature normally and do not impair early embryonic development [43]. On the other hand, mouse oocytes lacking *Dicer1* arrest in meiosis I due to disorganized spindles and defects in chromosomal alignment [44], [45]. This striking difference in phenotype between *Dicer1*- and *Dgcr8*-deficient oocytes suggests that endo-siRNAs, rather than miRNAs, are essential for oocyte maturation and the early stages of mammalian development [5].

Since miRNAs and endo-siRNAs act as posttranscriptional regulators of gene expression, the present study aims also at defining how the transcriptome of germ cells are affected in absence of these sncRNAs. For this purpose, we analyzed the transcriptional profiles of mice lacking *Dicer1* in pachytene spermatocytes by high throughput sequencing. Our results demonstrate that *Dicer1* inactivation of germ cell led to a global upregulation in gene expression. We observed that lowly expressed genes were most affected, compared to highly expressed genes. Interestingly, differentially expressed genes possess higher number of predicted miRNA target sites in their 3′UTR, suggesting that miRNAs function as post-transcriptional regulators by inducing mRNA degradation. Defects in tuning gene expression of target genes in differentiating male germ cells may affect their survival or the capacity to proceed to the meiotic and haploid phases of spermatogenesis. The functional implications of the observed gene expression changes were assessed by a gene ontology (GO) analysis and we identified 555 such GO categories. The categories with most significant enrichment included regulation of multicellular organismal process, genes linked to development, response to stimuli, and regulation of signaling and cell motility. Genes involved in regulation of apoptosis and cell death were also significantly enriched in GC-Dcr1 mutant spermatocytes (see **Table S3**). In contrast, genes related to spermatogenesis or meiosis were not found to be differentially expressed in the GC-Dcr1 mutants (data not shown). The large number of GO terms and the absence of terms related to spermatogenesis and meiosis makes it difficult to pinpoint specific pathways or biological processes causing the spermatogenic defects observed in GC-Dcr1 mutants. In fact, we believe that it is the large-scale upregulation of lowly expressed genes that affect as a whole the survival and differentiation process of mutant germ cells through cumulative impairments.

In conclusion, data from this study demonstrate that the core microprocessor component DGCR8, like DICER1, is essential for the meiotic and post-meiotic phases of spermatogenesis. Infertility, oligospermia and azoospermia, which are displayed by both GC-Dcr1 and GC-Dgcr8 mutants, likely represent the sum of the defects that accumulate during the different stages of spermatogenesis. The microprocessor complex DGCR8/DROSHA is essential for processing precursors of miRNAs, but is not required for endo-siRNA biogenesis. This allowed the phenotypic comparison between GC-Dgcr8 and GC-Dcr1 mutant mice to not only confirm that canonical miRNAs are essential in mediating meiotic and haploid phases of spermatogenesis but also to suggest that endo-siRNAs may potentially play a role in this process. Finally, transcriptomic analysis revealed that sncRNAs are important regulators of gene expression in spermatocytes, as they directly reduce the gene expression, especially for lowly expressed genes. Future studies aimed at addressing the functional role of individual miRNAs and endo-siRNAs could provide a deeper understanding of how these sncRNAs regulate the progression of germ cells through the meiotic and haploid phases of spermatogenesis.

## Material and Methods

### Animals

To achieve selective inactivation of *Dicer1* or *Dgcr8* in germ cells, we bred *Dcr1^flox^* (*Dcr1^fx/fx^*) [21] or *Dgcr8^flox^* (*Dgcr8^fx/fx^*) [22] with mice carrying a *Ddx4:Cre* (*Mvh:Cre*) [23] transgenic line, which expresses Cre recombinase under the control of the *Ddx4* promoter. All mice were genotyped by classic PCR as described previously [18], [22]. Animals were housed and cared for according to the ethical guidelines of the Direction Générale de la Santé of the Canton de Genève. All experimental procedures were approved by the Geneva Cantonal Veterinary Authority (autorization number: 1061/3698/1).

### Measurement of hormonal plasma levels

Blood was collected by cardiac puncture from adult mice at postnatal day 60 (P60). Plasma samples were stored at −20°C and were subsequently used to assess the levels of LH, FSH and testosterone. Hormone levels were measured in individual adult plasma samples (n = 3–9) from P60 adult mice. LH was measured by RIA, using a commercially available kit supplied by IDS (LH RIA CT # AHR002, Liège, Belgium), whereas FSH was measured by IRMA, using a kit from the same supplier (FSH IRMA CT # AHR004). Testosterone concentrations were assayed using a kit from MP Biomedicals (Testo DA kit, CT number 07-189102, Eschwege, Germany). Intra- and inter-assay coefficients of variation (CVs) in all three assays were less than 5% and 10%, respectively.

### Sperm count

Epididymal sperm count was performed on sperm extracted from the epididymis cauda and ductus deferens of adult (P60) male mice and was analyzed for its concentration as previously described [46].

### Histology and Immunofluorescence

Tissues were fixed overnight either in 4% PFA or in Bouin's fixative and embedded in paraffin. Five-mm sections were stained with haematoxylin and eosin (H&E) or processed for immunofluorescence (IF). For IF analysis, PFA-fixed sections were incubated overnight at 4°C with the following primary antibodies: anti- GATA4 (Santa Cruz Biotechnology, sc-9053, 1∶500), anti-protamine 1 (Shal technologies, Hup1N, 1∶1000), anti-pH3 (Ser10) (Millipore, Cat#06-570, 1∶500), anti-γH2AX (Calbiochem, Cat#dr-1017, 1∶500) and anti-H3K9me3 (Millipore, Cat#07-523, 1∶500). Alexa-conjugated secondary antibodies (Invitrogen) were then used for signal revelation and sections were counterstained using DAPI. All images were obtained with a Zeiss Axioscope microscope and processed using the ZEN software.

### Apoptosis Assays

Apoptotis assays were performed by TdTmediated X-dUTP nicked labeling (TUNEL) reactions using the Apoptag kit (Millipore); they were then stained with Permanent Red substrate-chromogen and counterstained with eosin. The percentage of apoptotic, TUNEL-positive cells was expressed as the average number of apoptotic cells within 20 seminiferous tubes. A minimum of 100 seminiferous tubules were counted per testis (5 sections/testis) and at least 3 animals per genotype per age were assessed.

### Electron microscopy

P60 testes from control, *Ddx4:Cre;Dgcr8^fx/fx^* and *Ddx4:Cre;Dcr1^fx/fx^* mutant individuals were fixed in 0.1 M sodium cacodylate with 4% gluteraldehyde in PBS. After fixation with 1% osmium tetroxide, the testes were embedded in epoxy resin (Glycidether 100, Merck). Selected areas were sectioned, stained with 5% uranyl acetate and 5% lead citrate, and visualized on a JEM-1400 Plus Transmission Electron Microscope equipped with OSIS Quemesa 11 Mpix bottom mounted digital camera.

### Pachytene spermatocyte isolation by centrifugal elutriation

Pachytene spermatocytes from *Ddx4:Cre;Dcr1^fx/fx^* mutant and control mice at P60 were purified by mechanical disruption and liberase treatment (Roche Applied Science). Cells were elutriated and separated into multiple fractions according to [47] and subsequently frozen for further analysis (n = 3, each pool of germ cells was isolated from either four pairs of *Ddx4:Cre;Dcr1^fx/fx^* mutant testes or two pairs of control testes). The purity of the pachytene spermatocytes was evaluated as previously described (Soumillon et al. 2013) based on fluorescence analysing using anti-SYCP3 (a marker for synaptonemal complex) and anti-phospho-H2AX (a marker for double-strand breaks and the sex body) and was estimated at ∼70%.

### RNA extraction, RNA-seq library preparation and Illumina sequencing

*RNA isolation*. Next generation sequencing of mRNA was performed on elutriated adult pachytene *Ddx4:Cre;Dcr1^fx/fx^* mutant and control spermatocytes according to Illumina's protocol using 200 ng of RNA. Total RNA was isolated using the Trizol protocol (Sigma-Aldrich). Genomic DNA was removed by DNase treatment. RNA integrity was measured using an Agilent 2100 bioanalyzer and samples had RIN >7.0.

*RNA-seq library preparation*. 200 ng of RNA was used to isolate polyA + RNA using Sera-Mag oligo(dT) beads (Thermo Scientific), fragmented with an NEBNext kit (BioLabs). First-strand cDNA was synthesized with random primers using the Superscript II polymerase (Invitrogen). Second strand cDNA, end-repair, A-base addition, and ligation of the Illumina PCR adaptators were performed according to Illumina's protocol. Libraries were then selected for 100 bp cDNA fragments on a 3.5% agarose gel and PCR-amplified using Phusion DNA polymerase (Finnzymes) for 15–18 cycles. PCR products were then purified on a 2% agarose gel and gel-extracted. Each library was quality controlled (product size and concentration) by an Agilent 2100 Bioanalyzer. Single-end libraries were sequenced as 100-mers on a Genome Analyzer II flowcell according to Illumina's protocol at a depth of ∼16.2–18.0 million reads per library.

### Gene expression analysis

RNA sequencing reads were aligned to the mouse genome (release mm10) using Bowtie2 2.1.0 and Tophat 2.0.10 with default settings [48], [49]. Expression levels were calculated as Fragments Per Kilobase of transcript per Million mapped reads (FPKM) using Cufflinks 2.1.1 [50]. To normalize the data, we identified 919 genes that did not contain predicted miRNA target sites, as identified by TargetScan release 6.2 [51] and were annotated as housekeeping genes [52], on the assumption that these genes would be the least likely to change their expression in the *Dicer1* knockout [53]. We then brought the median expression of these genes to a common value [54]. The expression level of each gene was determined as the median normalized log_2_-transformed FPKM value across the three replicates. Only genes that were annotated as protein-coding in Ensembl release 74 [55] and which were detected in all samples were kept for further analysis. All statistical analyses were carried out in R [56].

### Quantitative Real-Time PCR

For quantification in control, *Ddx4:Cre;Dgcr8^fx/fx^* and *Ddx4:Cre;Dcr1^fx/fx^* mutants, RNA from total testis extracts was isolated using Trizol (Sigma-Aldrich) according to the manufacturer's instructions. The mercury LNATM Universal RT microRNA PCR (Exiqon) was used for quantification of miRNAs according to the manufacturer's instructions. The relevant primers used are listed in **Table S4**. Data were analyzed using the 22DDCt method as described in [57]; U6 small RNA and 5S rRNA were used for miRNA level normalization. Each assay was performed in three independent technical and biological replicates.

### Statistical analysis

For the gene expression analysis, several nonparametric statistical analysis were performed including the Wilcoxon signed rank test, the Spearman's correlation coefficient and the Mann-Whitney test. All other results are expressed as means ± SEM of *n* experiments. The parametric unpaired *t* test was used for statistical analysis. Differences were considered statistically significant if *P* was <0.05.

## Supporting Information

Table S1 **Table comparing reproductive and endocrine measurements between GC-Dcr1 and GC-Dgcr8 mutants compared to control littermates.**
(PDF)

Table S2 **Enrichment analysis illustrating upregulated genes enriched for specific gene ontology (GO) terms in spermatocytes from GC-Dcr1 mutant mice.**
(PDF)

Table S3 **List of upregulated genes enriched in the GO term 0043067: regulation of programmed cell death, p value 1.79E-08.**
(PDF)

Table S4 **miRNAs primer sequences for real-time PCR.**
(PDF)
