# Supplementary material for: Germ Cell-Specific Targeting of DICER or DGCR8 Reveals a Novel Role for Endo-siRNAs in the Progression of Mammalian Spermatogenesis and Male Fertility
Source: PLoS One. 2014 Sep 22;9(9):e107023. doi: 10.1371/journal.pone.0107023 (PMC4171096; doi:10.1371/journal.pone.0107023)
Supplement: Table S1 — Table comparing reproductive and endocrine measurements between GC-Dcr1 and GC-Dgcr8 mutants compared to control littermates. (PDF) [file pone.0107023.s001.pdf]

Supplementary Table S1

P60 phenotype

Genotype	Control	GC-Dcr1		GC-Dgcr8	
Body weight (g.)	27.674 ± 1.200	29.245 ± 1.118	ns	26.042 ± 1.478	ns
Testes weight (g.)	0.212 ± 0.009	0.107 ± 0.007	***	0.097 ± 0.015	***
Epididymis weight (g.)	0.040 ± 0.002	0.031 ± 0.002	***	0.028 ± 0.002	***
Epididymal sperm conc. (10^6/ml)	10.040 ± 0.807	0.141 ± 0.041	***	0.319 ± 0.061	***
Seminal vesicles weight (g.)	0.216 ± 0.014	0.258 ± 0.019	ns	0.224 ± 0.021	ns
Tubule diameter (μm)	170.905 ± 1.716	122.517 ± 2.169	***	142.615 ± 1.813	***/#
Round spermatid number/tubule section	101.326 ± 3.251	40.854 ± 2.800	***	44.938 ± 3.160	***
Elongated spermatid number/tubule section	84.907 ± 1.030	21.052 ± 3.173	***	32.063 ± 1.270	***/#
Testerone plasma level (ng/ml)	1.089 ± 0.542	0.959 ± 0.466	ns	0.260 ± 0.140	ns

Values are the mean±SEM
*Significantly different from control mice, P<0.05
**Significantly different from control mice, P<0.001
***Significantly different from control mice, P<0.0001
#Significantly different between GC-Dcr1 and GC-Dgcr8, P<0.05
ns=not significative
