# Supplementary material for: Germ Cell-Specific Targeting of DICER or DGCR8 Reveals a Novel Role for Endo-siRNAs in the Progression of Mammalian Spermatogenesis and Male Fertility
Source: PLoS One. 2014 Sep 22;9(9):e107023. doi: 10.1371/journal.pone.0107023 (PMC4171096; doi:10.1371/journal.pone.0107023)
Supplement: Table S2 — Enrichment analysis illustrating upregulated genes enriched for specific gene ontology (GO) terms in spermatocytes from GC-Dcr1 mutant mice. (PDF) [file pone.0107023.s002.pdf]

Supplementary Table S2

Enrichment analysis illustrating upregulated and genes enriched for specific GO terms in pachytene spermatocytes from GC-Dcr1 mutant mice.

GO term	Description	P-value	FDR q-value	Number of genes
GO:0051239	regulation of multicellular organismal process	7.96E-26	8.58E-22	462
GO:0050896	response to stimulus	9.63E-25	5.19E-21	799
GO:0044699	single-organism process	6.56E-23	2.36E-19	1607
GO:0048518	positive regulation of biological process	1.37E-22	3.70E-19	775
GO:0065007	biological regulation	1.50E-22	3.22E-19	1581
GO:0044763	single-organism cellular process	7.76E-22	1.39E-18	1428
GO:0010033	response to organic substance	1.35E-21	2.07E-18	421
GO:0050789	regulation of biological process	1.98E-21	2.66E-18	1520
GO:0042221	response to chemical	2.40E-20	2.88E-17	542
GO:0006952	defense response	1.14E-19	1.23E-16	90
GO:0042127	regulation of cell proliferation	1.27E-19	1.25E-16	285
GO:0032501	multicellular organismal process	2.88E-19	2.59E-16	548
GO:0048522	positive regulation of cellular process	3.63E-19	3.01E-16	699
GO:0007165	signal transduction	4.72E-19	3.63E-16	533
GO:0050793	regulation of developmental process	5.26E-19	3.78E-16	388
GO:0007166	cell surface receptor signaling pathway	1.57E-18	1.06E-15	294
GO:0044707	single-multicellular organism process	1.66E-18	1.05E-15	539
GO:0022610	biological adhesion	2.94E-18	1.76E-15	173
GO:0051716	cellular response to stimulus	1.30E-17	7.38E-15	623
GO:0007155	cell adhesion	2.44E-17	1.31E-14	170
GO:0008284	positive regulation of cell proliferation	4.73E-17	2.43E-14	178
GO:0048584	positive regulation of response to stimulus	7.96E-17	3.90E-14	278
GO:0070887	cellular response to chemical stimulus	8.88E-17	4.16E-14	267
GO:0071310	cellular response to organic substance	1.15E-16	5.15E-14	230
GO:2000026	regulation of multicellular organismal development	5.67E-16	2.45E-13	273
GO:0050794	regulation of cellular process	6.93E-16	2.87E-13	1418
GO:0048513	organ development	7.78E-16	3.11E-13	242
GO:0002376	immune system process	1.38E-15	5.31E-13	166
GO:0048856	anatomical structure development	3.65E-15	1.36E-12	492
GO:0009719	response to endogenous stimulus	6.14E-15	2.21E-12	243
GO:0023056	positive regulation of signaling	1.01E-14	3.51E-12	242
GO:0051094	positive regulation of developmental process	1.03E-14	3.46E-12	204
GO:0006955	immune response	1.14E-14	3.72E-12	66
GO:0040017	positive regulation of locomotion	1.49E-14	4.72E-12	76
GO:0009653	anatomical structure morphogenesis	2.16E-14	6.67E-12	250
GO:0016477	cell migration	2.58E-14	7.71E-12	109
GO:0010647	positive regulation of cell communication	3.86E-14	1.13E-11	241
GO:0045595	regulation of cell differentiation	4.55E-14	1.29E-11	276
GO:0030335	positive regulation of cell migration	5.57E-14	1.54E-11	72
GO:0048583	regulation of response to stimulus	6.04E-14	1.63E-11	501
GO:0009607	response to biotic stimulus	7.96E-14	2.09E-11	66
GO:0048519	negative regulation of biological process	1.41E-13	3.61E-11	669
GO:2000147	positive regulation of cell motility	1.54E-13	3.85E-11	72
GO:0051240	positive regulation of multicellular organismal process	1.78E-13	4.35E-11	134
GO:0002682	regulation of immune system process	2.71E-13	6.50E-11	164
GO:0040012	regulation of locomotion	2.86E-13	6.70E-11	98
GO:0009967	positive regulation of signal transduction	2.94E-13	6.74E-11	229
GO:0051272	positive regulation of cellular component movement	3.19E-13	7.16E-11	73
GO:0009893	positive regulation of metabolic process	3.34E-13	7.35E-11	443
GO:0023051	regulation of signaling	5.10E-13	1.10E-10	452
GO:0032879	regulation of localization	6.86E-13	1.45E-10	256
GO:0051270	regulation of cellular component movement	7.26E-13	1.51E-10	170
GO:0030334	regulation of cell migration	7.65E-13	1.56E-10	90
GO:0010646	regulation of cell communication	7.93E-13	1.58E-10	452
GO:0045597	positive regulation of cell differentiation	1.16E-12	2.28E-10	148
GO:0032502	developmental process	1.22E-12	2.35E-10	741
GO:0044767	single-organism developmental process	1.27E-12	2.41E-10	691
GO:1901700	response to oxygen-containing compound	1.40E-12	2.60E-10	233
GO:0032101	regulation of response to external stimulus	2.28E-12	4.17E-10	47
GO:0010562	positive regulation of phosphorus metabolic process	3.08E-12	5.54E-10	178
GO:0045937	positive regulation of phosphate metabolic process	3.08E-12	5.45E-10	178
GO:0040011	locomotion	3.96E-12	6.88E-10	121
GO:0009611	response to wounding	4.02E-12	6.88E-10	88
GO:2000145	regulation of cell motility	4.06E-12	6.85E-10	106
GO:1902533	positive regulation of intracellular signal transduction	4.16E-12	6.89E-10	145
GO:0051704	multi-organism process	1.18E-11	1.93E-09	69

GO:0009966	regulation of signal transduction	1.71E-11	2.76E-09	365
GO:0048729	tissue morphogenesis	1.74E-11	2.76E-09	102
GO:0031325	positive regulation of cellular metabolic process	1.80E-11	2.81E-09	423
GO:0006950	response to stress	2.13E-11	3.28E-09	550
GO:0014070	response to organic cyclic compound	2.46E-11	3.73E-09	150
GO:0052547	regulation of peptidase activity	3.90E-11	5.85E-09	110
GO:0009725	response to hormone	4.11E-11	6.07E-09	138
GO:0065008	regulation of biological quality	4.93E-11	7.18E-09	349
GO:0051241	negative regulation of multicellular organismal process	5.00E-11	7.18E-09	75
GO:0051707	response to other organism	6.04E-11	8.57E-09	45
GO:0048870	cell motility	6.57E-11	9.20E-09	113
GO:0010604	positive regulation of macromolecule metabolic process	7.75E-11	1.07E-08	397
GO:0042327	positive regulation of phosphorylation	8.13E-11	1.11E-08	159
GO:0050678	regulation of epithelial cell proliferation	9.87E-11	1.33E-08	36
GO:0048523	negative regulation of cellular process	1.03E-10	1.37E-08	595
GO:0043410	positive regulation of MAPK cascade	1.26E-10	1.66E-08	89
GO:0048869	cellular developmental process	1.37E-10	1.78E-08	373
GO:0033993	response to lipid	1.45E-10	1.86E-08	128
GO:0009888	tissue development	1.79E-10	2.27E-08	112
GO:0022603	regulation of anatomical structure morphogenesis	2.10E-10	2.63E-08	166
GO:0010628	positive regulation of gene expression	2.21E-10	2.73E-08	261
GO:0098542	defense response to other organism	2.26E-10	2.77E-08	38
GO:0030278	regulation of ossification	2.76E-10	3.34E-08	46
GO:0002684	positive regulation of immune system process	3.21E-10	3.85E-08	84
GO:0030154	cell differentiation	4.59E-10	5.44E-08	279
GO:0002009	morphogenesis of an epithelium	7.45E-10	8.73E-08	78
GO:0051173	positive regulation of nitrogen compound metabolic process	1.02E-09	1.19E-07	281
GO:1901701	cellular response to oxygen-containing compound	1.13E-09	1.30E-07	116
GO:0030155	regulation of cell adhesion	1.45E-09	1.64E-07	51
GO:0045087	innate immune response	1.68E-09	1.89E-07	37
GO:0051049	regulation of transport	1.74E-09	1.94E-07	184
GO:0007167	enzyme linked receptor protein signaling pathway	2.15E-09	2.36E-07	109
GO:0006629	lipid metabolic process	2.28E-09	2.48E-07	217
GO:0031347	regulation of defense response	2.65E-09	2.85E-07	48
GO:0042742	defense response to bacterium	2.71E-09	2.90E-07	23
GO:0045935	positive regulation of nucleobase-containing compound metabolic process	3.01E-09	3.18E-07	274
GO:0006928	cellular component movement	3.07E-09	3.21E-07	141
GO:0009891	positive regulation of biosynthetic process	3.18E-09	3.30E-07	288
GO:0031328	positive regulation of cellular biosynthetic process	3.63E-09	3.72E-07	284
GO:0050776	regulation of immune response	3.75E-09	3.81E-07	54
GO:0071495	cellular response to endogenous stimulus	4.08E-09	4.11E-07	125
GO:0006954	inflammatory response	4.80E-09	4.79E-07	62
GO:0030324	lung development	5.83E-09	5.77E-07	36
GO:0010941	regulation of cell death	6.11E-09	5.99E-07	270
GO:0050679	positive regulation of epithelial cell proliferation	6.17E-09	5.99E-07	24
GO:0050731	positive regulation of peptidyl-tyrosine phosphorylation	1.03E-08	9.90E-07	41
GO:0030198	extracellular matrix organization	1.05E-08	1.00E-06	52
GO:0034097	response to cytokine	1.12E-08	1.06E-06	64
GO:0007186	G-protein coupled receptor signaling pathway	1.17E-08	1.10E-06	56
GO:0048660	regulation of smooth muscle cell proliferation	1.21E-08	1.13E-06	30
GO:1901698	response to nitrogen compound	1.24E-08	1.14E-06	157
GO:0009617	response to bacterium	1.55E-08	1.42E-06	18
GO:0043062	extracellular structure organization	1.57E-08	1.42E-06	52
GO:0010243	response to organonitrogen compound	1.62E-08	1.45E-06	130
GO:0050730	regulation of peptidyl-tyrosine phosphorylation	1.77E-08	1.58E-06	55
GO:0043067	regulation of programmed cell death	1.79E-08	1.58E-06	314
GO:0019882	antigen processing and presentation	1.91E-08	1.68E-06	21
GO:0009887	organ morphogenesis	2.25E-08	1.96E-06	92
GO:0010557	positive regulation of macromolecule biosynthetic process	2.28E-08	1.97E-06	266
GO:0035456	response to interferon-beta	2.77E-08	2.37E-06	7
GO:0051050	positive regulation of transport	2.78E-08	2.36E-06	160
GO:0035556	intracellular signal transduction	2.97E-08	2.50E-06	223
GO:0032844	regulation of homeostatic process	3.36E-08	2.81E-06	40
GO:0042325	regulation of phosphorylation	3.38E-08	2.80E-06	222
GO:0001817	regulation of cytokine production	3.43E-08	2.82E-06	51
GO:0048731	system development	3.68E-08	3.00E-06	130
GO:0043408	regulation of MAPK cascade	3.70E-08	3.00E-06	118
GO:0001932	regulation of protein phosphorylation	3.89E-08	3.13E-06	184
GO:1902531	regulation of intracellular signal transduction	4.12E-08	3.29E-06	225
GO:0048589	developmental growth	4.33E-08	3.43E-06	49
GO:0018212	peptidyl-tyrosine modification	4.40E-08	3.46E-06	43

GO:0051246	regulation of protein metabolic process	4.62E-08	3.61E-06	171
GO:0010035	response to inorganic substance	4.66E-08	3.62E-06	74
GO:0048661	positive regulation of smooth muscle cell proliferation	4.76E-08	3.66E-06	23
GO:0061138	morphogenesis of a branching epithelium	5.09E-08	3.89E-06	48
GO:0001934	positive regulation of protein phosphorylation	5.46E-08	4.15E-06	128
GO:0044057	regulation of system process	5.51E-08	4.15E-06	125
GO:0008283	cell proliferation	6.24E-08	4.67E-06	39
GO:0051093	negative regulation of developmental process	6.26E-08	4.65E-06	139
GO:0060548	negative regulation of cell death	6.27E-08	4.63E-06	146
GO:0080134	regulation of response to stress	6.38E-08	4.68E-06	87
GO:0018108	peptidyl-tyrosine phosphorylation	6.44E-08	4.69E-06	42
GO:0071216	cellular response to biotic stimulus	6.78E-08	4.90E-06	17
GO:0010466	negative regulation of peptidase activity	7.10E-08	5.11E-06	26
GO:0002237	response to molecule of bacterial origin	7.84E-08	5.60E-06	23
GO:0048878	chemical homeostasis	8.26E-08	5.86E-06	60
GO:0051254	positive regulation of RNA metabolic process	8.65E-08	6.10E-06	242
GO:0048545	response to steroid hormone	1.02E-07	7.11E-06	65
GO:0001525	angiogenesis	1.04E-07	7.22E-06	64
GO:0008217	regulation of blood pressure	1.06E-07	7.34E-06	30
GO:0008202	steroid metabolic process	1.13E-07	7.79E-06	35
GO:0042981	regulation of apoptotic process	1.17E-07	7.98E-06	319
GO:0016337	cell-cell adhesion	1.23E-07	8.34E-06	76
GO:0050778	positive regulation of immune response	1.34E-07	9.04E-06	39
GO:0050830	defense response to Gram-positive bacterium	1.50E-07	1.01E-05	12
GO:0065009	regulation of molecular function	1.52E-07	1.01E-05	210
GO:0045893	positive regulation of transcription, DNA-templated	1.58E-07	1.05E-05	233
GO:0048585	negative regulation of response to stimulus	1.61E-07	1.06E-05	272
GO:0050818	regulation of coagulation	1.76E-07	1.15E-05	15
GO:0050829	defense response to Gram-negative bacterium	1.84E-07	1.19E-05	6
GO:0001763	morphogenesis of a branching structure	1.94E-07	1.25E-05	28
GO:0001568	blood vessel development	2.26E-07	1.45E-05	20
GO:0009605	response to external stimulus	2.56E-07	1.63E-05	90
GO:0071219	cellular response to molecule of bacterial origin	2.62E-07	1.66E-05	15
GO:0010810	regulation of cell-substrate adhesion	2.65E-07	1.67E-05	28
GO:1900046	regulation of hemostasis	3.14E-07	1.97E-05	13
GO:0030193	regulation of blood coagulation	3.14E-07	1.96E-05	13
GO:0050865	regulation of cell activation	3.38E-07	2.09E-05	99
GO:0052548	regulation of endopeptidase activity	3.56E-07	2.19E-05	85
GO:0045944	positive regulation of transcription from RNA polymerase II promoter	3.61E-07	2.21E-05	174
GO:0001775	cell activation	3.76E-07	2.29E-05	73
GO:0097485	neuron projection guidance	3.95E-07	2.39E-05	34
GO:0007411	axon guidance	3.95E-07	2.38E-05	34
GO:0070167	regulation of biomineral tissue development	4.00E-07	2.39E-05	24
GO:0050920	regulation of chemotaxis	4.14E-07	2.47E-05	19
GO:0006935	chemotaxis	4.49E-07	2.66E-05	12
GO:0002253	activation of immune response	4.64E-07	2.73E-05	31
GO:0050795	regulation of behavior	4.70E-07	2.75E-05	40
GO:0042330	taxis	4.91E-07	2.86E-05	12
GO:0071396	cellular response to lipid	5.32E-07	3.09E-05	57
GO:0042476	odontogenesis	5.65E-07	3.26E-05	22
GO:0003008	system process	6.44E-07	3.69E-05	166
GO:0050727	regulation of inflammatory response	6.75E-07	3.85E-05	24
GO:0071345	cellular response to cytokine stimulus	7.22E-07	4.10E-05	41
GO:0050790	regulation of catalytic activity	7.52E-07	4.25E-05	181
GO:0009968	negative regulation of signal transduction	8.39E-07	4.71E-05	233
GO:0051336	regulation of hydrolase activity	8.50E-07	4.75E-05	82
GO:0019221	cytokine-mediated signaling pathway	1.01E-06	5.60E-05	30
GO:0002252	immune effector process	1.19E-06	6.55E-05	37
GO:1901615	organic hydroxy compound metabolic process	1.19E-06	6.52E-05	78
GO:0023057	negative regulation of signaling	1.27E-06	6.94E-05	243
GO:0045596	negative regulation of cell differentiation	1.32E-06	7.18E-05	148
GO:0048754	branching morphogenesis of an epithelial tube	1.33E-06	7.19E-05	41
GO:0031290	retinal ganglion cell axon guidance	1.48E-06	7.97E-05	6
GO:0002521	leukocyte differentiation	1.54E-06	8.23E-05	39
GO:0051051	negative regulation of transport	1.61E-06	8.59E-05	65
GO:0050900	leukocyte migration	1.66E-06	8.80E-05	16
GO:0051248	negative regulation of protein metabolic process	1.69E-06	8.93E-05	67
GO:0019222	regulation of metabolic process	1.72E-06	9.02E-05	915
GO:0009987	cellular process	1.88E-06	9.84E-05	1965
GO:0048771	tissue remodeling	1.89E-06	9.86E-05	24
GO:0055082	cellular chemical homeostasis	2.01E-06	1.04E-04	46

GO:0006875	cellular metal ion homeostasis	2.02E-06	1.04E-04	42
GO:0061041	regulation of wound healing	2.03E-06	1.04E-04	15
GO:0010951	negative regulation of endopeptidase activity	2.09E-06	1.07E-04	19
GO:0048002	antigen processing and presentation of peptide antigen	2.10E-06	1.07E-04	16
GO:0043085	positive regulation of catalytic activity	2.24E-06	1.13E-04	97
GO:0010648	negative regulation of cell communication	2.26E-06	1.14E-04	243
GO:0051346	negative regulation of hydrolase activity	2.47E-06	1.24E-04	31
GO:0044093	positive regulation of molecular function	2.61E-06	1.31E-04	223
GO:0007169	transmembrane receptor protein tyrosine kinase signaling pathway	2.62E-06	1.30E-04	66
GO:0043069	negative regulation of programmed cell death	2.84E-06	1.40E-04	130
GO:0051249	regulation of lymphocyte activation	2.95E-06	1.45E-04	78
GO:0050870	positive regulation of T cell activation	2.99E-06	1.46E-04	30
GO:0007610	behavior	3.08E-06	1.50E-04	99
GO:0001503	ossification	3.10E-06	1.50E-04	34
GO:0050819	negative regulation of coagulation	3.12E-06	1.51E-04	9
GO:0007275	multicellular organismal development	3.14E-06	1.51E-04	134
GO:0045321	leukocyte activation	3.16E-06	1.51E-04	106
GO:0032653	regulation of interleukin-10 production	3.24E-06	1.55E-04	8
GO:0070372	regulation of ERK1 and ERK2 cascade	3.75E-06	1.78E-04	36
GO:0051174	regulation of phosphorus metabolic process	3.91E-06	1.85E-04	148
GO:0042592	homeostatic process	4.04E-06	1.90E-04	160
GO:0046777	protein autophosphorylation	4.32E-06	2.03E-04	57
GO:0006874	cellular calcium ion homeostasis	4.39E-06	2.05E-04	31
GO:0031349	positive regulation of defense response	4.50E-06	2.09E-04	24
GO:0006357	regulation of transcription from RNA polymerase II promoter	4.79E-06	2.21E-04	292
GO:0070663	regulation of leukocyte proliferation	4.93E-06	2.27E-04	50
GO:0048146	positive regulation of fibroblast proliferation	4.96E-06	2.28E-04	13
GO:0042509	regulation of tyrosine phosphorylation of STAT protein	4.99E-06	2.28E-04	14
GO:0019220	regulation of phosphate metabolic process	5.09E-06	2.32E-04	147
GO:0048812	neuron projection morphogenesis	5.11E-06	2.31E-04	44
GO:0044281	small molecule metabolic process	5.18E-06	2.34E-04	315
GO:1902107	positive regulation of leukocyte differentiation	5.24E-06	2.35E-04	27
GO:0051047	positive regulation of secretion	5.25E-06	2.35E-04	21
GO:0070206	protein trimerization	5.63E-06	2.51E-04	11
GO:0040007	growth	5.88E-06	2.61E-04	71
GO:0050921	positive regulation of chemotaxis	5.94E-06	2.62E-04	15
GO:0043066	negative regulation of apoptotic process	6.01E-06	2.65E-04	126
GO:0045785	positive regulation of cell adhesion	6.03E-06	2.64E-04	50
GO:0030500	regulation of bone mineralization	6.03E-06	2.63E-04	21
GO:0044092	negative regulation of molecular function	6.24E-06	2.71E-04	129
GO:0006937	regulation of muscle contraction	6.39E-06	2.77E-04	24
GO:0030595	leukocyte chemotaxis	6.40E-06	2.76E-04	12
GO:0006690	icosanoid metabolic process	6.85E-06	2.94E-04	9
GO:0033559	unsaturated fatty acid metabolic process	6.85E-06	2.93E-04	9
GO:1901568	fatty acid derivative metabolic process	6.85E-06	2.92E-04	9
GO:0070208	protein heterotrimerization	7.20E-06	3.06E-04	6
GO:0010811	positive regulation of cell-substrate adhesion	7.23E-06	3.06E-04	19
GO:0002694	regulation of leukocyte activation	7.60E-06	3.20E-04	87
GO:0008285	negative regulation of cell proliferation	7.85E-06	3.29E-04	105
GO:0055114	oxidation-reduction process	7.86E-06	3.28E-04	83
GO:0048598	embryonic morphogenesis	8.07E-06	3.36E-04	68
GO:0032787	monocarboxylic acid metabolic process	8.32E-06	3.45E-04	43
GO:0042531	positive regulation of tyrosine phosphorylation of STAT protein	8.62E-06	3.56E-04	11
GO:0048520	positive regulation of behavior	8.78E-06	3.61E-04	27
GO:1901617	organic hydroxy compound biosynthetic process	8.82E-06	3.62E-04	42
GO:0032103	positive regulation of response to external stimulus	8.84E-06	3.61E-04	20
GO:0046649	lymphocyte activation	8.91E-06	3.62E-04	86
GO:0048608	reproductive structure development	9.00E-06	3.65E-04	47
GO:0090257	regulation of muscle system process	9.30E-06	3.75E-04	34
GO:0006082	organic acid metabolic process	9.53E-06	3.83E-04	166
GO:0006873	cellular ion homeostasis	9.78E-06	3.92E-04	36
GO:0044255	cellular lipid metabolic process	9.80E-06	3.91E-04	162
GO:0002573	myeloid leukocyte differentiation	1.00E-05	3.99E-04	16
GO:0002764	immune response-regulating signaling pathway	1.02E-05	4.03E-04	25
GO:1901342	regulation of vasculature development	1.05E-05	4.16E-04	25
GO:0030003	cellular cation homeostasis	1.05E-05	4.15E-04	35
GO:0051960	regulation of nervous system development	1.07E-05	4.20E-04	176
GO:0034754	cellular hormone metabolic process	1.09E-05	4.28E-04	12
GO:0002687	positive regulation of leukocyte migration	1.10E-05	4.26E-04	12
GO:0071223	cellular response to lipoteichoic acid	1.12E-05	4.34E-04	4
GO:0070391	response to lipoteichoic acid	1.12E-05	4.32E-04	4

GO:0045926	negative regulation of growth	1.20E-05	4.64E-04	17
GO:0060341	regulation of cellular localization	1.22E-05	4.68E-04	129
GO:0010959	regulation of metal ion transport	1.25E-05	4.80E-04	34
GO:0034109	homotypic cell-cell adhesion	1.29E-05	4.93E-04	16
GO:0050801	ion homeostasis	1.32E-05	5.00E-04	122
GO:0055074	calcium ion homeostasis	1.32E-05	5.01E-04	31
GO:0034341	response to interferon-gamma	1.34E-05	5.07E-04	7
GO:0001819	positive regulation of cytokine production	1.39E-05	5.23E-04	37
GO:0007389	pattern specification process	1.41E-05	5.28E-04	82
GO:0060601	lateral sprouting from an epithelium	1.42E-05	5.30E-04	6
GO:0080090	regulation of primary metabolic process	1.47E-05	5.47E-04	797
GO:0010955	negative regulation of protein processing	1.50E-05	5.57E-04	9
GO:0060326	cell chemotaxis	1.54E-05	5.69E-04	15
GO:0097190	apoptotic signaling pathway	1.56E-05	5.73E-04	82
GO:0072503	cellular divalent inorganic cation homeostasis	1.63E-05	5.99E-04	31
GO:0008016	regulation of heart contraction	1.68E-05	6.13E-04	32
GO:0032944	regulation of mononuclear cell proliferation	1.71E-05	6.23E-04	48
GO:0044708	single-organism behavior	1.75E-05	6.36E-04	77
GO:0055065	metal ion homeostasis	1.79E-05	6.47E-04	103
GO:0051128	regulation of cellular component organization	1.87E-05	6.73E-04	265
GO:0010817	regulation of hormone levels	2.03E-05	7.30E-04	29
GO:0002685	regulation of leukocyte migration	2.04E-05	7.29E-04	14
GO:0032836	glomerular basement membrane development	2.05E-05	7.32E-04	9
GO:0007507	heart development	2.08E-05	7.38E-04	67
GO:0007154	cell communication	2.11E-05	7.48E-04	99
GO:0051247	positive regulation of protein metabolic process	2.16E-05	7.64E-04	93
GO:0032870	cellular response to hormone stimulus	2.17E-05	7.65E-04	68
GO:0042445	hormone metabolic process	2.17E-05	7.63E-04	15
GO:0071363	cellular response to growth factor stimulus	2.34E-05	8.19E-04	60
GO:0001764	neuron migration	2.35E-05	8.19E-04	28
GO:0002673	regulation of acute inflammatory response	2.42E-05	8.41E-04	12
GO:0045088	regulation of innate immune response	2.42E-05	8.40E-04	22
GO:0010952	positive regulation of peptidase activity	2.48E-05	8.57E-04	50
GO:0032352	positive regulation of hormone metabolic process	2.49E-05	8.58E-04	10
GO:0060644	mammary gland epithelial cell differentiation	2.52E-05	8.65E-04	6
GO:0019369	arachidonic acid metabolic process	2.53E-05	8.67E-04	10
GO:1900047	negative regulation of hemostasis	2.57E-05	8.76E-04	8
GO:0030195	negative regulation of blood coagulation	2.57E-05	8.73E-04	8
GO:0045940	positive regulation of steroid metabolic process	2.58E-05	8.74E-04	15
GO:0042110	T cell activation	2.58E-05	8.72E-04	39
GO:0050867	positive regulation of cell activation	2.60E-05	8.75E-04	49
GO:1901724	positive regulation of cell proliferation involved in kidney developmen	2.64E-05	8.87E-04	4
GO:0003006	developmental process involved in reproduction	2.68E-05	8.97E-04	83
GO:0071222	cellular response to lipopolysaccharide	2.69E-05	8.97E-04	13
GO:0060021	palate development	2.75E-05	9.15E-04	35
GO:0070374	positive regulation of ERK1 and ERK2 cascade	2.76E-05	9.16E-04	22
GO:0010517	regulation of phospholipase activity	2.78E-05	9.19E-04	10
GO:0008406	gonad development	2.78E-05	9.17E-04	33
GO:0008610	lipid biosynthetic process	2.84E-05	9.32E-04	101
GO:0002920	regulation of humoral immune response	2.88E-05	9.42E-04	10
GO:0070848	response to growth factor	2.88E-05	9.39E-04	62
GO:0050767	regulation of neurogenesis	2.96E-05	9.63E-04	148
GO:0006631	fatty acid metabolic process	2.97E-05	9.63E-04	32
GO:0006820	anion transport	2.97E-05	9.63E-04	57
GO:0051251	positive regulation of lymphocyte activation	3.00E-05	9.69E-04	55
GO:0002221	pattern recognition receptor signaling pathway	3.04E-05	9.78E-04	13
GO:0002758	innate immune response-activating signal transduction	3.04E-05	9.75E-04	13
GO:0050670	regulation of lymphocyte proliferation	3.15E-05	1.01E-03	47
GO:0043436	oxoacid metabolic process	3.24E-05	1.03E-03	161
GO:0010863	positive regulation of phospholipase C activity	3.39E-05	1.08E-03	7
GO:0050810	regulation of steroid biosynthetic process	3.50E-05	1.11E-03	28
GO:0071407	cellular response to organic cyclic compound	3.62E-05	1.14E-03	54
GO:0051222	positive regulation of protein transport	3.65E-05	1.15E-03	54
GO:0072089	stem cell proliferation	3.79E-05	1.19E-03	11
GO:0019216	regulation of lipid metabolic process	3.82E-05	1.20E-03	60
GO:0001558	regulation of cell growth	3.90E-05	1.22E-03	43
GO:0031589	cell-substrate adhesion	3.93E-05	1.22E-03	35
GO:0040008	regulation of growth	4.19E-05	1.30E-03	31
GO:0009880	embryonic pattern specification	4.62E-05	1.43E-03	15
GO:0002696	positive regulation of leukocyte activation	4.62E-05	1.43E-03	58
GO:2000021	regulation of ion homeostasis	4.77E-05	1.47E-03	20

GO:0048646	anatomical structure formation involved in morphogenesis	4.80E-05	1.47E-03	153
GO:0090031	positive regulation of steroid hormone biosynthetic process	4.80E-05	1.47E-03	7
GO:0045669	positive regulation of osteoblast differentiation	4.90E-05	1.50E-03	19
GO:0008210	estrogen metabolic process	4.98E-05	1.52E-03	8
GO:0071621	granulocyte chemotaxis	5.06E-05	1.54E-03	7
GO:0097530	granulocyte migration	5.06E-05	1.53E-03	7
GO:0032268	regulation of cellular protein metabolic process	5.18E-05	1.56E-03	134
GO:0016125	sterol metabolic process	5.20E-05	1.57E-03	31
GO:0019725	cellular homeostasis	5.52E-05	1.66E-03	89
GO:0001562	response to protozoan	5.62E-05	1.68E-03	7
GO:0071417	cellular response to organonitrogen compound	5.78E-05	1.73E-03	53
GO:0072507	divalent inorganic cation homeostasis	6.00E-05	1.79E-03	31
GO:0001936	regulation of endothelial cell proliferation	6.06E-05	1.80E-03	14
GO:0001667	ameboidal cell migration	6.14E-05	1.82E-03	32
GO:0050878	regulation of body fluid levels	6.16E-05	1.82E-03	29
GO:0048732	gland development	6.18E-05	1.82E-03	33
GO:0070665	positive regulation of leukocyte proliferation	6.50E-05	1.91E-03	36
GO:0032490	detection of molecule of bacterial origin	6.52E-05	1.91E-03	4
GO:0042129	regulation of T cell proliferation	6.54E-05	1.91E-03	29
GO:0032970	regulation of actin filament-based process	6.74E-05	1.96E-03	52
GO:0045124	regulation of bone resorption	6.92E-05	2.01E-03	8
GO:0055080	cation homeostasis	7.05E-05	2.04E-03	38
GO:0006979	response to oxidative stress	7.32E-05	2.12E-03	76
GO:0019752	carboxylic acid metabolic process	7.42E-05	2.14E-03	147
GO:0010975	regulation of neuron projection development	7.52E-05	2.16E-03	101
GO:0002688	regulation of leukocyte chemotaxis	7.58E-05	2.17E-03	10
GO:0012501	programmed cell death	7.62E-05	2.18E-03	217
GO:0010875	positive regulation of cholesterol efflux	7.69E-05	2.19E-03	5
GO:0051223	regulation of protein transport	7.84E-05	2.23E-03	89
GO:0022602	ovulation cycle process	7.86E-05	2.23E-03	10
GO:0033674	positive regulation of kinase activity	7.90E-05	2.23E-03	48
GO:0051480	cytosolic calcium ion homeostasis	7.93E-05	2.24E-03	19
GO:0042475	odontogenesis of dentin-containing tooth	8.14E-05	2.29E-03	18
GO:0042517	positive regulation of tyrosine phosphorylation of Stat3 protein	8.27E-05	2.32E-03	8
GO:0030449	regulation of complement activation	8.30E-05	2.32E-03	7
GO:0090030	regulation of steroid hormone biosynthetic process	8.59E-05	2.40E-03	10
GO:0002504	antigen processing and presentation of peptide or polysaccharide antigen via MHC class II	8.68E-05	2.42E-03	8
GO:0002495	antigen processing and presentation of peptide antigen via MHC class II	8.68E-05	2.41E-03	8
GO:0019934	cGMP-mediated signaling	8.82E-05	2.44E-03	4
GO:0060997	dendritic spine morphogenesis	9.06E-05	2.51E-03	7
GO:0045834	positive regulation of lipid metabolic process	9.16E-05	2.52E-03	41
GO:0002690	positive regulation of leukocyte chemotaxis	9.18E-05	2.53E-03	9
GO:1901564	organonitrogen compound metabolic process	9.20E-05	2.52E-03	277
GO:2001237	negative regulation of extrinsic apoptotic signaling pathway	9.23E-05	2.52E-03	40
GO:0035234	ectopic germ cell programmed cell death	9.29E-05	2.54E-03	8
GO:0070169	positive regulation of biomineral tissue development	9.33E-05	2.54E-03	13
GO:0046886	positive regulation of hormone biosynthetic process	9.74E-05	2.64E-03	9
GO:0046425	regulation of JAK-STAT cascade	1.01E-04	2.73E-03	10
GO:0043405	regulation of MAP kinase activity	1.01E-04	2.73E-03	58
GO:0070613	regulation of protein processing	1.02E-04	2.74E-03	14
GO:0060600	dichotomous subdivision of an epithelial terminal unit	1.02E-04	2.74E-03	7
GO:0060255	regulation of macromolecule metabolic process	1.06E-04	2.83E-03	1084
GO:0045860	positive regulation of protein kinase activity	1.06E-04	2.83E-03	45
GO:0060736	prostate gland growth	1.06E-04	2.83E-03	7
GO:0010720	positive regulation of cell development	1.07E-04	2.85E-03	39
GO:2001233	regulation of apoptotic signaling pathway	1.07E-04	2.85E-03	121
GO:0002757	immune response-activating signal transduction	1.08E-04	2.87E-03	22
GO:0031344	regulation of cell projection organization	1.09E-04	2.89E-03	75
GO:0043086	negative regulation of catalytic activity	1.15E-04	3.02E-03	99
GO:0055085	transmembrane transport	1.15E-04	3.02E-03	189
GO:0031399	regulation of protein modification process	1.16E-04	3.05E-03	208
GO:0007204	positive regulation of cytosolic calcium ion concentration	1.17E-04	3.07E-03	18
GO:0031401	positive regulation of protein modification process	1.18E-04	3.07E-03	72
GO:1900274	regulation of phospholipase C activity	1.18E-04	3.08E-03	7
GO:0051046	regulation of secretion	1.21E-04	3.14E-03	28
GO:0032946	positive regulation of mononuclear cell proliferation	1.22E-04	3.15E-03	35
GO:0071902	positive regulation of protein serine/threonine kinase activity	1.24E-04	3.21E-03	32
GO:0050771	negative regulation of axonogenesis	1.24E-04	3.20E-03	7
GO:0002697	regulation of immune effector process	1.29E-04	3.31E-03	28
GO:1901722	regulation of cell proliferation involved in kidney development	1.29E-04	3.32E-03	7
GO:0090287	regulation of cellular response to growth factor stimulus	1.30E-04	3.34E-03	36

GO:2001234	negative regulation of apoptotic signaling pathway	1.35E-04	3.44E-03	65
GO:0001938	positive regulation of endothelial cell proliferation	1.35E-04	3.44E-03	23
GO:0010893	positive regulation of steroid biosynthetic process	1.36E-04	3.45E-03	12
GO:0051347	positive regulation of transferase activity	1.38E-04	3.49E-03	91
GO:0010950	positive regulation of endopeptidase activity	1.41E-04	3.58E-03	44
GO:0022407	regulation of cell-cell adhesion	1.41E-04	3.57E-03	30
GO:0060670	branching involved in labyrinthine layer morphogenesis	1.42E-04	3.58E-03	4
GO:0070201	regulation of establishment of protein localization	1.45E-04	3.64E-03	100
GO:0032846	positive regulation of homeostatic process	1.45E-04	3.65E-03	13
GO:0060325	face morphogenesis	1.46E-04	3.64E-03	9
GO:0045664	regulation of neuron differentiation	1.47E-04	3.67E-03	122
GO:0060560	developmental growth involved in morphogenesis	1.47E-04	3.67E-03	28
GO:0009890	negative regulation of biosynthetic process	1.48E-04	3.69E-03	291
GO:0060284	regulation of cell development	1.49E-04	3.69E-03	173
GO:0001816	cytokine production	1.51E-04	3.72E-03	19
GO:0035458	cellular response to interferon-beta	1.52E-04	3.74E-03	5
GO:0042832	defense response to protozoan	1.53E-04	3.76E-03	6
GO:0050890	cognition	1.55E-04	3.81E-03	48
GO:0032388	positive regulation of intracellular transport	1.57E-04	3.84E-03	53
GO:0097529	myeloid leukocyte migration	1.58E-04	3.86E-03	9
GO:0006811	ion transport	1.59E-04	3.88E-03	139
GO:0001501	skeletal system development	1.63E-04	3.97E-03	31
GO:0071634	regulation of transforming growth factor beta production	1.64E-04	3.98E-03	9
GO:0044283	small molecule biosynthetic process	1.64E-04	3.98E-03	97
GO:0019229	regulation of vasoconstriction	1.67E-04	4.03E-03	14
GO:0002921	negative regulation of humoral immune response	1.68E-04	4.05E-03	6
GO:0032269	negative regulation of cellular protein metabolic process	1.74E-04	4.19E-03	107
GO:0032386	regulation of intracellular transport	1.76E-04	4.22E-03	95
GO:0032496	response to lipopolysaccharide	1.77E-04	4.23E-03	23
GO:0071346	cellular response to interferon-gamma	1.80E-04	4.31E-03	6
GO:0042523	positive regulation of tyrosine phosphorylation of Stat5 protein	1.84E-04	4.38E-03	5
GO:0009889	regulation of biosynthetic process	1.85E-04	4.41E-03	836
GO:0046890	regulation of lipid biosynthetic process	1.89E-04	4.48E-03	47
GO:0043269	regulation of ion transport	1.89E-04	4.49E-03	88
GO:0045933	positive regulation of muscle contraction	1.90E-04	4.50E-03	3
GO:1901699	cellular response to nitrogen compound	1.94E-04	4.57E-03	54
GO:0045765	regulation of angiogenesis	1.94E-04	4.57E-03	17
GO:0010518	positive regulation of phospholipase activity	1.97E-04	4.62E-03	8
GO:0008219	cell death	1.98E-04	4.64E-03	227
GO:0050863	regulation of T cell activation	1.98E-04	4.63E-03	52
GO:0032989	cellular component morphogenesis	2.04E-04	4.76E-03	90
GO:2000257	regulation of protein activation cascade	2.06E-04	4.79E-03	7
GO:1902105	regulation of leukocyte differentiation	2.06E-04	4.80E-03	37
GO:0002218	activation of innate immune response	2.09E-04	4.86E-03	13
GO:0045089	positive regulation of innate immune response	2.14E-04	4.94E-03	16
GO:0043523	regulation of neuron apoptotic process	2.14E-04	4.94E-03	65
GO:0043277	apoptotic cell clearance	2.16E-04	4.96E-03	9
GO:0032956	regulation of actin cytoskeleton organization	2.18E-04	5.01E-03	47
GO:0050671	positive regulation of lymphocyte proliferation	2.29E-04	5.26E-03	34
GO:0045778	positive regulation of ossification	2.29E-04	5.25E-03	15
GO:0035815	positive regulation of renal sodium excretion	2.30E-04	5.25E-03	3
GO:0044706	multi-multicellular organism process	2.31E-04	5.26E-03	7
GO:0050715	positive regulation of cytokine secretion	2.32E-04	5.27E-03	9
GO:0032373	positive regulation of sterol transport	2.33E-04	5.29E-03	5
GO:0032376	positive regulation of cholesterol transport	2.33E-04	5.28E-03	5
GO:0071383	cellular response to steroid hormone stimulus	2.33E-04	5.27E-03	28
GO:0045667	regulation of osteoblast differentiation	2.34E-04	5.28E-03	26
GO:0060907	positive regulation of macrophage cytokine production	2.41E-04	5.43E-03	3
GO:0006584	catecholamine metabolic process	2.42E-04	5.42E-03	13
GO:0009712	catechol-containing compound metabolic process	2.42E-04	5.41E-03	13
GO:0042516	regulation of tyrosine phosphorylation of Stat3 protein	2.42E-04	5.42E-03	10
GO:0022604	regulation of cell morphogenesis	2.43E-04	5.42E-03	113
GO:0032270	positive regulation of cellular protein metabolic process	2.44E-04	5.44E-03	81
GO:0016265	death	2.47E-04	5.50E-03	229
GO:0044087	regulation of cellular component biogenesis	2.48E-04	5.49E-03	85
GO:0009892	negative regulation of metabolic process	2.48E-04	5.50E-03	328
GO:0044711	single-organism biosynthetic process	2.54E-04	5.62E-03	216
GO:0032963	collagen metabolic process	2.58E-04	5.68E-03	10
GO:0032652	regulation of interleukin-1 production	2.58E-04	5.67E-03	10
GO:0035455	response to interferon-alpha	2.60E-04	5.70E-03	7
GO:0002604	regulation of dendritic cell antigen processing and presentation	2.71E-04	5.94E-03	3

GO:0010942	positive regulation of cell death	2.77E-04	6.05E-03	128
GO:0048514	blood vessel morphogenesis	2.77E-04	6.05E-03	21
GO:2001056	positive regulation of cysteine-type endopeptidase activity	2.77E-04	6.04E-03	41
GO:0030099	myeloid cell differentiation	2.78E-04	6.04E-03	23
GO:0045916	negative regulation of complement activation	2.82E-04	6.12E-03	5
GO:2000258	negative regulation of protein activation cascade	2.82E-04	6.11E-03	5
GO:0071229	cellular response to acid	2.83E-04	6.11E-03	13
GO:0010605	negative regulation of macromolecule metabolic process	2.90E-04	6.25E-03	304
GO:0032655	regulation of interleukin-12 production	2.90E-04	6.25E-03	9
GO:0032680	regulation of tumor necrosis factor production	2.91E-04	6.25E-03	15
GO:2001236	regulation of extrinsic apoptotic signaling pathway	2.92E-04	6.25E-03	43
GO:0000302	response to reactive oxygen species	2.96E-04	6.33E-03	34
GO:0006940	regulation of smooth muscle contraction	2.97E-04	6.34E-03	14
GO:0042522	regulation of tyrosine phosphorylation of Stat5 protein	2.97E-04	6.33E-03	6
GO:0019932	second-messenger-mediated signaling	3.00E-04	6.38E-03	11
GO:0030501	positive regulation of bone mineralization	3.06E-04	6.49E-03	12
GO:0045766	positive regulation of angiogenesis	3.15E-04	6.68E-03	29
GO:0030316	osteoclast differentiation	3.25E-04	6.87E-03	8
GO:0072126	positive regulation of glomerular mesangial cell proliferation	3.31E-04	6.98E-03	3
GO:0007229	integrin-mediated signaling pathway	3.34E-04	7.04E-03	21
GO:0001894	tissue homeostasis	3.41E-04	7.17E-03	18
GO:0010631	epithelial cell migration	3.43E-04	7.20E-03	20
GO:0051923	sulfation	3.43E-04	7.19E-03	2
GO:0032651	regulation of interleukin-1 beta production	3.44E-04	7.18E-03	8
GO:0060429	epithelium development	3.46E-04	7.21E-03	20
GO:0048145	regulation of fibroblast proliferation	3.47E-04	7.21E-03	14
GO:0046850	regulation of bone remodeling	3.55E-04	7.37E-03	9
GO:0051057	positive regulation of small GTPase mediated signal transduction	3.58E-04	7.42E-03	15
GO:0001822	kidney development	3.58E-04	7.41E-03	40
GO:0019884	antigen processing and presentation of exogenous antigen	3.58E-04	7.40E-03	11
GO:0048546	digestive tract morphogenesis	3.59E-04	7.40E-03	9
GO:0019218	regulation of steroid metabolic process	3.60E-04	7.40E-03	25
GO:0032760	positive regulation of tumor necrosis factor production	3.61E-04	7.41E-03	4
GO:0044710	single-organism metabolic process	3.63E-04	7.44E-03	491
GO:0090092	regulation of transmembrane receptor protein serine/threonine kinase signaling pathway	3.68E-04	7.53E-03	38
GO:0042102	positive regulation of T cell proliferation	3.74E-04	7.64E-03	21
GO:2001012	mesenchymal cell differentiation involved in renal system development	3.75E-04	7.65E-03	2
GO:0072161	mesenchymal cell differentiation involved in kidney development	3.75E-04	7.64E-03	2
GO:0072162	metanephric mesenchymal cell differentiation	3.75E-04	7.62E-03	2
GO:0022029	telencephalon cell migration	3.77E-04	7.65E-03	4
GO:0070098	chemokine-mediated signaling pathway	3.79E-04	7.66E-03	5
GO:0034755	iron ion transmembrane transport	3.87E-04	7.82E-03	9
GO:0022414	reproductive process	4.06E-04	8.18E-03	54
GO:0019886	antigen processing and presentation of exogenous peptide antigen via MHC class II	4.07E-04	8.19E-03	7
GO:0006959	humoral immune response	4.12E-04	8.26E-03	13
GO:0006066	alcohol metabolic process	4.17E-04	8.36E-03	55
GO:0072124	regulation of glomerular mesangial cell proliferation	4.24E-04	8.49E-03	5
GO:0014874	response to stimulus involved in regulation of muscle adaptation	4.31E-04	8.61E-03	2
GO:0042088	T-helper 1 type immune response	4.35E-04	8.67E-03	5
GO:0010613	positive regulation of cardiac muscle hypertrophy	4.39E-04	8.73E-03	11
GO:0014742	positive regulation of muscle hypertrophy	4.39E-04	8.71E-03	11
GO:0001676	long-chain fatty acid metabolic process	4.39E-04	8.70E-03	19
GO:0006665	sphingolipid metabolic process	4.46E-04	8.82E-03	32
GO:0048568	embryonic organ development	4.47E-04	8.83E-03	35
GO:1901214	regulation of neuron death	4.51E-04	8.89E-03	69
GO:0042510	regulation of tyrosine phosphorylation of Stat1 protein	4.55E-04	8.94E-03	6
GO:0044259	multicellular organismal macromolecule metabolic process	4.55E-04	8.94E-03	10
GO:0046579	positive regulation of Ras protein signal transduction	4.61E-04	9.04E-03	13
GO:0048638	regulation of developmental growth	4.70E-04	9.19E-03	52
GO:0030855	epithelial cell differentiation	4.79E-04	9.35E-03	69
GO:0046427	positive regulation of JAK-STAT cascade	4.79E-04	9.33E-03	12
GO:0034698	response to gonadotropin	4.80E-04	9.35E-03	10
GO:0044703	multi-organism reproductive process	4.89E-04	9.50E-03	7
