# Supplementary material for: Germ Cell-Specific Targeting of DICER or DGCR8 Reveals a Novel Role for Endo-siRNAs in the Progression of Mammalian Spermatogenesis and Male Fertility
Source: PLoS One. 2014 Sep 22;9(9):e107023. doi: 10.1371/journal.pone.0107023 (PMC4171096; doi:10.1371/journal.pone.0107023)
Supplement: Table S3 — List of upregulated genes enriched in the GO term 0043067: regulation of programmed cell death, p value 1.79E-08. (PDF) [file pone.0107023.s003.pdf]

Supplementary Table S3

Upregulated genes enriched in the GO term 0043067: regulation of programmed cell death, p value 1.79E-08

Gene symbol	Gene name/description	Expression values in control log2(FPKM)	Expression values in GC-Dcr1 log2(FPKM)	Upregulation
Acaa2	acetyl coenzyme a acyltransferase 2 (mitochondrial 3 oxoacyl coenzyme a thiolase)	0.573	2.312	1.739
Acer2	alkaline ceramidase 2	0.496	2.032	1.535
Acvr1	activin a receptor, type 1	-2.250	-0.904	1.346
Ada	adenosine deaminase	-0.529	0.365	0.894
Adar	adenosine deaminase, rna specific	-0.827	0.583	1.410
Aes	amino terminal enhancer of split	1.847	3.042	1.195
Agrn	agrin	-1.679	-0.269	1.410
Agt	angiotensinogen (serpin peptidase inhibitor, clade a, member 8)	-2.097	0.983	3.080
Aifm1	apoptosis inducing factor, mitochondrion associated 1	-0.151	0.422	0.573
Aldh1a1	aldehyde dehydrogenase family 1, subfamily a1	3.138	4.833	1.695
Aldh1a3	aldehyde dehydrogenase family 1, subfamily a3	-3.426	-0.861	2.566
Aldh2	aldehyde dehydrogenase 2, mitochondrial	1.547	3.023	1.476
Alkbh1	alkb, alkylation repair homolog 1 (e. coli)	2.110	2.664	0.554
Alox12	arachidonate 12 lipoxygenase	-2.081	-1.374	0.707
Anxa1	annexin a1	0.594	1.624	1.030
Anxa5	annexin a5	-0.139	1.571	1.710
Apaf1	apoptotic peptidase activating factor 1	-1.422	-0.355	1.067
Apoe	apolipoprotein e	2.991	4.780	1.789
Ar	androgen receptor	-1.835	-0.728	1.107
Asns	asparagine synthetase	6.139	6.898	0.759
Atf4	activating transcription factor 4	3.341	4.226	0.885
Atf5	activating transcription factor 5	1.577	2.131	0.554
Atg5	autophagy related 5	0.017	0.859	0.842
Atm	ataxia telangiectasia mutated homolog (human)	-0.234	1.382	1.616
Atp7a	atpase, cu++ transporting, alpha polypeptide	-1.335	-0.422	0.913
Axl	axl receptor tyrosine kinase	1.330	2.128	0.797
B4galt1	udp gal:betaglcnac beta 1,4 galactosyltransferase, polypeptide 1	1.310	2.084	0.774
Bax	bcl2 associated x protein	0.403	1.018	0.615
Bcap31	b cell receptor associated protein 31	2.123	2.916	0.794
Bcl10	b cell leukemia/lymphoma 10	0.922	1.513	0.591
Bcl2a1b	b cell leukemia/lymphoma 2 related protein a1b	-4.055	-1.653	2.403
Bcl2l2	bcl2 like 2	0.312	1.234	0.922
Bcl3	b cell leukemia/lymphoma 3	-1.827	-0.294	1.532
Bclaf1	bcl2 associated transcription factor 1	3.531	4.150	0.619
Bdkrb2	bradykinin receptor, beta 2	-3.989	-3.368	0.621
Bid	bh3 interacting domain death agonist	-0.905	0.206	1.111
Bmp7	bone morphogenetic protein 7	-1.859	-0.890	0.968
Btg2	b cell translocation gene 2, anti proliferative	-0.159	1.952	2.110
C6	complement component 6	-1.637	-0.279	1.359
Card11	caspase recruitment domain family, member 11	-4.643	-3.751	0.892
Casp2	caspase 2	0.252	1.665	1.412
Casp7	caspase 7	-2.413	-0.819	1.594
Casp8ap2	caspase 8 associated protein 2	1.094	1.829	0.735
Casp9	caspase 9	0.277	1.071	0.794
Cat	catalase	-0.273	0.589	0.862
Cav1	caveolin 1, caveolae protein	-1.175	0.585	1.760
Ccng1	cyclin g1	-0.129	1.230	1.359
Ccr7	chemokine (c c motif) receptor 7	-0.267	1.290	1.557
Cd248	cd248 antigen, endosialin	-1.062	0.092	1.154
Cd24a	cd24a antigen	1.076	1.775	0.699
Cd59b	cd59b antigen	4.083	5.375	1.292
Cd74	cd74 antigen (invariant polypeptide of major histocompatibility complex, class ii antigen associated)	0.059	1.969	1.910
Cdc42	cell division cycle 42	4.065	4.762	0.697
Cdkn1b	cyclin dependent kinase inhibitor 1b	-1.670	-0.548	1.122

Cdkn2a	cyclin dependent kinase inhibitor 2	-0.683	0.092	0.774
Cflar	casp8 and fadd like apoptosis regulator	-0.688	0.377	1.065
Cited1	cbp/p300 interacting transactivator with glu/asp rich carboxy terminal domain 1	2.572	3.198	0.626
Cited2	cbp/p300 interacting transactivator, with glu/asp rich carboxy terminal domain, 2	0.105	0.764	0.659
Clip3	cap gly domain containing linker protein 3	-0.791	1.284	2.076
Cln8	ceroid lipofuscinosis, neuronal 8	-2.973	-2.186	0.787
Clu	clusterin	5.435	6.647	1.212
Col18a1	collagen, type xviii, alpha 1	-2.567	-1.834	0.733
Col4a3	collagen, type iv, alpha 3	-1.736	-0.373	1.363
Cpeb4	cytoplasmic polyadenylation element binding protein 4	4.332	4.894	0.563
Creb1	camp responsive element binding protein 1	0.354	0.990	0.636
Cryab	crystallin, alpha b	-3.513	-0.857	2.655
Csrnp3	cysteine serine rich nuclear protein 3	2.921	3.616	0.695
Cst3	cystatin c	4.554	5.476	0.922
Ctgf	connective tissue growth factor	0.341	2.426	2.085
Ctnna1	catenin (cadherin associated protein), alpha 1	1.714	2.654	0.940
Ctsc	cathepsin c	-1.718	-0.311	1.407
Ctsh	cathepsin h	-1.743	0.502	2.245
Cul7	cullin 7	-0.792	0.243	1.035
Cxcr4	chemokine (c x c motif) receptor 4	-4.291	-0.898	3.393
Cycs	cytochrome c, somatic	3.140	3.790	0.650
Cyld	cylindromatosis (turban tumor syndrome)	0.251	1.163	0.912
Cyr61	cysteine rich protein 61	0.130	1.355	1.225
Dab2	disabled 2, mitogen responsive phosphoprotein	-1.661	0.357	2.019
Dapk1	death associated protein kinase 1	-4.202	-2.262	1.940
Ddx3x	dead/h (asp glu ala asp/his) box polypeptide 3, xlinked	1.475	3.193	1.718
Deptor	dep domain containing mtor interacting protein	-3.870	-2.014	1.856
Dffa	dna fragmentation factor, alpha subunit	0.565	1.258	0.693
Dhodh	dihydroorotate dehydrogenase	-1.238	1.177	2.416
Dlc1	deleted in liver cancer 1	-3.081	-1.116	1.966
Dnm1l	dynamin 1 like	4.436	5.140	0.704
Dusp1	dual specificity phosphatase 1	1.863	2.663	0.800
Dusp2	dual specificity phosphatase 2	-0.555	0.512	1.067
Ednra	endothelin receptor type a	-0.301	0.639	0.940
Egfr	epidermal growth factor receptor	-1.268	-0.276	0.992
Egln3	egl nine homolog 3 (c. elegans)	-2.248	-1.489	0.759
Egr1	early growth response 1	2.402	4.483	2.081
Egr2	early growth response 2	-2.928	-1.440	1.488
Egr3	early growth response 3	1.645	2.207	0.562
Egr4	early growth response 4	-1.295	-0.450	0.845
Eif2ak3	eukaryotic translation initiation factor 2 alpha kinase 3	-4.138	-2.359	1.778
Ell3	elongation factor rna polymerase ii like 3	-0.629	0.841	1.470
Erbb2	v erb b2 erythroblastic leukemia viral oncogene homolog 2, neuro/glioblastoma derived oncogene homolog (avian)	-1.682	-0.841	0.841
Erbb3	v erb b2 erythroblastic leukemia viral oncogene homolog 3	-2.829	-1.585	1.244
Esr1	estrogen receptor 1 (alpha)	-2.429	-0.305	2.124
F3	coagulation factor iii	-0.928	0.527	1.455
Fam129b	family with sequence similarity 129, member b	-1.285	-0.420	0.866
Fgfr1	fibroblast growth factor receptor 1	-0.473	0.238	0.711
Fgfr3	fibroblast growth factor receptor 3	-1.628	-0.716	0.912
Fhl2	four and a half lim domains 2	0.298	1.952	1.654
Fn1	fibronectin 1	0.175	0.870	0.694
Foxo1	forkhead box o1	-1.069	-0.167	0.902
Foxo3	forkhead box o3	-0.088	0.744	0.832
Fyn	fyn proto oncogene	1.050	1.986	0.935
G6pdx	glucose 6 phosphate dehydrogenase x linked	-0.327	0.475	0.802
Gabrb3	gamma aminobutyric acid (gaba) a receptor, subunit beta 3	-1.154	-0.569	0.585
Gadd45b	growth arrest and dna damage inducible 45 beta	0.693	1.979	1.286
Gadd45g	growth arrest and dna damage inducible 45 gamma	0.652	1.893	1.240
Gas1	growth arrest specific 1	-3.382	-1.859	1.523
Gas6	growth arrest specific 6	0.790	1.833	1.043
Gata1	gata binding protein 1	-1.045	0.003	1.048
Gata3	gata binding protein 3	-2.993	-1.832	1.162
Gata6	gata binding protein 6	0.224	1.167	0.943

Gli3	gli kruppel family member gli3	-3.162	-2.123	1.039
Gls2	glutaminase 2 (liver, mitochondrial)	-1.819	-0.464	1.355
Gnaq	guanine nucleotide binding protein, alpha q polypeptide	-1.456	0.150	1.607
Gnrh1	gonadotropin releasing hormone 1	-1.837	-1.200	0.637
Gpam	glycerol 3 phosphate acyltransferase, mitochondrial	-0.797	-0.209	0.588
Gstp1	glutathione s transferase, pi 1	0.045	0.667	0.622
Hck	hemopoietic cell kinase	-4.431	-2.764	1.667
Hdac2	histone deacetylase 2	3.933	4.500	0.567
Hdac6	histone deacetylase 6	1.392	2.282	0.889
Hells	helicase, lymphoid specific	3.483	4.112	0.629
Hey2	hairy/enhancer of split related with yrpw motif 2	-3.965	-1.797	2.167
Hif1a	hypoxia inducible factor 1, alpha subunit	0.368	1.445	1.078
Hmgb2	high mobility group box 2	4.442	5.147	0.706
Hmox1	heme oxygenase (decycling) 1	0.322	1.253	0.931
Hspb1	heat shock protein 1	1.392	2.132	0.739
Id3	inhibitor of dna binding 3	-1.811	0.162	1.973
Ier3	immediate early response 3	-0.175	1.385	1.560
Ifi27	interferon, alpha inducible protein 27	1.831	2.417	0.586
Igbp1	immunoglobulin (cd79a) binding protein 1	-1.000	-0.443	0.557
Igf1	insulin like growth factor 1	1.061	2.707	1.646
Igf1r	insulin like growth factor i receptor	-1.047	-0.121	0.926
Igf2r	insulin like growth factor 2 receptor	-0.742	0.645	1.387
Il1b	interleukin 1 beta	-0.720	0.021	0.741
Inhbb	inhibin beta b	-1.496	-0.941	0.555
Insl3	insulin like 3	3.367	5.566	2.199
Irs2	insulin receptor substrate 2	-1.294	-0.734	0.559
Itga5	integrin alpha 5 (fibronectin receptor alpha)	-5.141	-2.335	2.805
Itga6	integrin alpha 6	1.382	2.097	0.715
Itgav	integrin alpha v	-2.679	-1.004	1.674
Itgb1	integrin beta 1 (fibronectin receptor beta)	1.801	3.094	1.293
Jak2	janus kinase 2	-0.981	0.209	1.190
Jun	jun oncogene	1.339	2.796	1.456
Kank2	kn motif and ankyrin repeat domains 2	-1.265	-0.601	0.664
Kcnh8	potassium voltage gated channel, subfamily h (eag related), member 8	1.145	1.895	0.750
Kit	kit oncogene	-0.260	0.951	1.211
Kitl	kit ligand	-0.720	0.601	1.321
Klf11	kruppel like factor 11	-1.569	-0.553	1.017
Klhl20	kelch like 20	1.045	1.663	0.618
Kras	v ki ras2 kirsten rat sarcoma viral oncogene homolog	3.056	3.815	0.758
Lck	lymphocyte protein tyrosine kinase	-1.243	1.792	3.034
Lgmn	legumain	1.796	2.916	1.120
Lmna	lamin a	2.214	2.801	0.587
Lpar1	lysophosphatidic acid receptor 1	3.696	4.260	0.564
Lrp1	low density lipoprotein receptor related protein 1	-0.636	1.273	1.908
Lrp6	low density lipoprotein receptor related protein 6	-1.458	0.002	1.460
Ltbr	lymphotoxin b receptor	0.545	1.300	0.755
Maged1	melanoma antigen, family d, 1	4.150	4.762	0.612
Malt1	mucosa associated lymphoid tissue lymphoma translocation gene 1	0.174	1.117	0.942
Map2k4	mitogen activated protein kinase kinase 4	0.394	1.688	1.294
Mapk7	mitogen activated protein kinase 7	-0.038	0.832	0.870
Mdk	midkine	0.940	1.677	0.737
Mdm4	transformed mouse 3t3 cell double minute 4	4.910	5.680	0.770
Melk	maternal embryonic leucine zipper kinase	-0.996	0.624	1.620
Met	met proto oncogene	-4.275	-2.875	1.400
Mgmt	o 6 methylguanine dna methyltransferase	-1.050	0.256	1.307
Mical1	microtubule associated monooxygenase, calponin and lim domain containing 1	-6.173	-4.417	1.756
Mitf	microphthalmia associated transcription factor	-3.802	-3.191	0.612
Mmp2	matrix metallopeptidase 2	-2.993	-0.840	2.153
Msh2	muts homolog 2 (e. coli)	1.193	2.084	0.892
Myc	myelocytomatosis oncogene	-2.186	-0.115	2.071
Nacc2	nucleus accumbens associated 2	-2.314	-1.332	0.983
Nae1	nedd8 activating enzyme e1 subunit 1	4.313	4.941	0.628
Naip1	nlr family, apoptosis inhibitory protein 1	-6.985	-5.612	1.373

Nanos3	nanos homolog 3 (drosophila)	-3.884	-2.467	1.417
Ncf2	neutrophil cytosolic factor 2	-3.990	-2.207	1.784
Ndufaf4	nadh dehydrogenase (ubiquinone) 1 alpha subcomplex, assembly factor 4	0.150	0.888	0.738
Nes	nestin	-4.332	-3.418	0.914
Nfe2l2	nuclear factor, erythroid derived 2, like 2	1.859	2.603	0.744
Nle1	notchless homolog 1 (drosophila)	-0.334	0.647	0.981
Nos2	nitric oxide synthase 2, inducible	-4.670	-2.767	1.903
Notch2	notch 2	-1.464	-0.599	0.865
Nr4a1	nuclear receptor subfamily 4, group a, member 1	3.742	4.493	0.752
Nr4a2	nuclear receptor subfamily 4, group a, member 2	-3.509	-1.521	1.988
Nr4a3	nuclear receptor subfamily 4, group a, member 3	-1.638	-0.453	1.185
Nrbp2	nuclear receptor binding protein 2	-0.738	0.201	0.939
Nrp1	neuropilin 1	-3.365	-0.303	3.062
Nsmf	nmda receptor synaptonuclear signaling and neuronal migration factor	2.761	3.680	0.919
Ntrk2	neurotrophic tyrosine kinase, receptor, type 2	-1.736	-0.587	1.149
Nuak2	nuak family, snf1 like kinase, 2	-2.245	-1.108	1.137
Osr1	odd skipped related 1 (drosophila)	-5.423	-2.791	2.632
P2rx7	purinergic receptor p2x, ligand gated ion channel, 7	-1.806	-0.827	0.979
P4hb	prolyl 4 hydroxylase, beta polypeptide	3.924	4.953	1.029
Pak3	p21 protein (cdc42/rac) activated kinase 3	3.613	4.203	0.589
Pak7	p21 protein (cdc42/rac) activated kinase 7	-6.172	-3.073	3.098
Palb2	partner and localizer of brca2	1.040	1.711	0.671
Pcgf2	polycomb group ring finger 2	-3.248	-0.585	2.663
Pdcd6	programmed cell death 6	2.666	3.298	0.632
Pde5a	phosphodiesterase 5a, cgmp specific	-2.867	-2.013	0.854
Pea15a	phosphoprotein enriched in astrocytes 15a	1.063	2.155	1.092
Pf4	platelet factor 4	-3.550	-0.907	2.644
Pgap2	post gpi attachment to proteins 2	5.402	5.979	0.577
Phip	pleckstrin homology domain interacting protein	0.150	1.036	0.886
Phlda1	pleckstrin homology like domain, family a, member 1	-4.567	-3.175	1.392
Pik3r1	phosphatidylinositol 3 kinase, regulatory subunit, polypeptide 1 (p85 alpha)	-2.245	-1.482	0.763
Plagl2	pleiomorphic adenoma gene like 2	2.644	3.235	0.592
Plcg2	phospholipase c, gamma 2	0.771	2.162	1.391
Pmaip1	phorbol 12 myristate 13 acetate induced protein 1	-0.778	0.332	1.109
Pnp	purine nucleoside phosphorylase	0.767	1.383	0.616
Por	p450 (cytochrome) oxidoreductase	1.839	2.608	0.769
Ppard	peroxisome proliferator activator receptor delta	-3.104	-0.820	2.284
Pparg	peroxisome proliferator activated receptor gamma	-0.995	0.311	1.306
Ppif	peptidylprolyl isomerase f (cyclophilin f)	0.591	1.228	0.637
Ppm1f	protein phosphatase 1f (pp2c domain containing)	1.723	2.603	0.881
Ppp1ca	protein phosphatase 1, catalytic subunit, alpha isoform	1.049	1.915	0.866
Prkaa2	protein kinase, amp activated, alpha 2 catalytic subunit	-4.017	-2.561	1.456
Prkca	protein kinase c, alpha	-1.129	-0.168	0.961
Prkcg	protein kinase c, gamma	-5.372	-4.131	1.240
Prkci	protein kinase c, iota	0.805	1.393	0.588
Prkdc	protein kinase, dna activated, catalytic polypeptide	5.413	5.130	-0.283
Prnp	prion protein	0.933	1.728	0.795
Psen2	presenilin 2	-2.955	-2.147	0.808
Psmd10	proteasome (prosome, macropain) 26s subunit, non atpase, 10	3.143	3.837	0.694
Psme3	proteaseome (prosome, macropain) activator subunit 3 (pa28 gamma, ki)	6.503	7.161	0.658
Ptprc	protein tyrosine phosphatase, receptor type, c	-3.468	-1.525	1.943
Rag1	recombination activating gene 1	-3.070	-2.374	0.695
Ramp2	receptor (calcitonin) activity modifying protein 2	-2.350	-1.626	0.724
Rapgef2	rap guanine nucleotide exchange factor (gef) 2	-0.749	0.167	0.915
Rarg	retinoic acid receptor, gamma	-1.114	-0.324	0.790
Rhbdd1	rhomboid domain containing 1	4.017	4.578	0.561
Rhob	ras homolog gene family, member b	0.857	1.866	1.009
Ripk2	receptor (tnfrsf) interacting serine threonine kinase 2	-1.518	-0.325	1.193
Robo1	roundabout homolog 1 (drosophila)	-0.925	-0.048	0.877
Rps6ka2	ribosomal protein s6 kinase, polypeptide 2	0.197	0.770	0.574
Rps6kb1	ribosomal protein s6 kinase, polypeptide 1	0.878	1.904	1.026

Rrm2b	ribonucleotide reductase m2 b (tp53 inducible)	-1.544	-0.542	1.002
Rrn3	rrn3 rna polymerase i transcription factor homolog (yeast)	1.922	2.637	0.715
S100b	s100 protein, beta polypeptide, neural	-2.015	-0.581	1.434
Serinc3	serine incorporator 3	5.722	6.345	0.623
Serpine1	serine (or cysteine) peptidase inhibitor, clade e, member 1	-2.375	-0.645	1.730
Sgk1	serum/glucocorticoid regulated kinase 1	-2.497	-1.220	1.277
Sh3rf1	sh3 domain containing ring finger 1	-2.688	-1.917	0.771
Siglec1	sialic acid binding ig like lectin 1, sialoadhesin	-3.705	-2.391	1.314
Sik1	salt inducible kinase 1	1.204	2.647	1.442
Sirt1	sirtuin 1	2.952	3.570	0.619
Siva1	siva1, apoptosis inducing factor	2.772	3.346	0.574
Slc11a2	solute carrier family 11 (proton coupled divalent metal ion transporters), member 2	1.625	2.231	0.606
Slc25a27	solute carrier family 25, member 27	-2.261	-0.653	1.608
Slc25a5	solute carrier family 25 (mitochondrial carrier, adenine nucleotide translocator), member 5	1.914	3.180	1.265
Slc40a1	solute carrier family 40 (iron regulated transporter), member 1	-3.233	-2.209	1.024
Slit2	slit homolog 2 (drosophila)	-3.435	-2.412	1.023
Smad3	smad family member 3	-2.835	-1.343	1.492
Smo	smoothened homolog (drosophila)	-1.922	-0.950	0.972
Smpd2	sphingomyelin phosphodiesterase 2, neutral	2.042	2.895	0.853
Snai1	snail homolog 1 (drosophila)	-0.665	0.376	1.041
Sox10	sry box containing gene 10	-0.992	-0.154	0.839
Spdef	sam pointed domain containing ets transcription factor	-0.839	0.334	1.173
Sphk1	sphingosine kinase 1	-5.181	-2.008	3.173
Spry2	sprouty homolog 2 (drosophila)	-2.784	-1.114	1.670
Src	rous sarcoma oncogene	-1.510	-0.728	0.782
Star	steroidogenic acute regulatory protein	-0.070	1.201	1.271
Steap3	steap family member 3	-2.157	-1.177	0.980
Stk4	serine/threonine kinase 4	3.204	3.930	0.726
Sycp2	synaptonemal complex protein 2	6.319	7.120	0.801
Syvn1	synovial apoptosis inhibitor 1, synoviolin	2.633	3.264	0.631
Tbx1	t box 1	-0.690	0.909	1.600
Tcf7l2	transcription factor 7 like 2, t cell specific, hmg box	-2.246	-0.297	1.949
Terf1	telomeric repeat binding factor 1	3.919	4.516	0.597
Tex11	testis expressed gene 11	1.283	2.040	0.756
Tfap4	transcription factor ap4	-2.397	-1.711	0.686
Tgfb3	transforming growth factor, beta 3	-0.431	0.161	0.592
Tgfbr1	transforming growth factor, beta receptor i	3.940	4.761	0.821
Tgm2	transglutaminase 2, c polypeptide	-1.876	-0.263	1.613
Thbs1	thrombospondin 1	-1.876	-0.263	1.613
Tle1	transducin like enhancer of split 1, homolog of drosophila e(spl)	-3.793	-2.157	1.635
Tmem132a	transmembrane protein 132a	-4.974	-2.728	2.246
Tmem161a	transmembrane protein 161a	0.084	0.687	0.602
Tnfaip3	tumor necrosis factor, alpha induced protein 3	-0.062	1.474	1.535
Tnfaip8	tumor necrosis factor, alpha induced protein 8	-5.353	-2.384	2.968
Tnfrsf1a	tumor necrosis factor receptor superfamily, member 1a	-0.329	0.368	0.696
Tnip2	tnfaip3 interacting protein 2	-1.035	-0.250	0.785
Tpd52l1	tumor protein d52 like 1	0.169	1.030	0.861
Tpt1	tumor protein, translationally controlled 1	8.984	9.640	0.656
Traf1	tnf receptor associated factor 1	0.428	2.223	1.795
Traf3	tnf receptor associated factor 3	-1.598	-0.425	1.173
Traf4	tnf receptor associated factor 4	-0.373	1.121	1.493
Traf6	tnf receptor associated factor 6	-3.612	-2.410	1.202
Triap1	tp53 regulated inhibitor of apoptosis 1	0.990	1.578	0.588
Trim2	tripartite motif containing 2	-5.168	-3.888	1.280
Trp63	transformation related protein 63	-2.101	-1.071	1.030
Trp73	transformation related protein 73	-4.736	-3.643	1.093
Tsc22d3	tsc22 domain family, member 3	1.143	2.040	0.897
Tspo	translocator protein	-1.837	-0.512	1.324
Txnip	thioredoxin interacting protein	3.261	3.948	0.686
Tyro3	tyro3 protein tyrosine kinase 3	-0.001	0.834	0.835
Ucp2	uncoupling protein 2 (mitochondrial, proton carrier)	1.749	2.337	0.588
Unc5b	unc 5 homolog b (c. elegans)	-3.993	-3.393	0.600

Ung	uracil dna glycosylase	-0.562	0.527	1.088
Vegfa	vascular endothelial growth factor a	-3.992	-2.365	1.627
Wnt3a	wingless related mmtv integration site 3a	-1.218	-0.489	0.729
Wt1	wilms tumor 1 homolog	0.674	1.532	0.858
Xdh	xanthine dehydrogenase	-3.096	-0.459	2.637
Xiap	x linked inhibitor of apoptosis	-1.705	-0.760	0.945
Xrcc2	x ray repair complementing defective repair in chinese hamster cells 2	2.633	3.263	0.630
Zbtb16	zinc finger and btb domain containing 16	-2.466	-1.407	1.059
Zfp622	zinc finger protein 622	1.017	1.602	0.585
