# Supplementary material for: Germ Cell-Specific Targeting of DICER or DGCR8 Reveals a Novel Role for Endo-siRNAs in the Progression of Mammalian Spermatogenesis and Male Fertility
Source: PLoS One. 2014 Sep 22;9(9):e107023. doi: 10.1371/journal.pone.0107023 (PMC4171096; doi:10.1371/journal.pone.0107023)
Supplement: Table S4 — miRNAs primer sequences for real-time PCR. (PDF) [file pone.0107023.s004.pdf]

Supp. Table S4: Primers Sequences for Real-Time PCR

Transcripts	Forward 5' → 3'
**miRNAs**	
**mmu-miR-34c-3p**	AAUCACUAACCACACAGCCAGG
**hsa-miR-184-3p**	UGGACGGAGAACUGAUAAGGGU
**mmu-miR-376a-3p**	AUCGUAGAGGAAAAUCCACGU
